# Competing Magnetism in Layered Mixed Transition Metal Chalcogenides KCo_2–*x*_Ni_*x*_Se_2_, KCo_2–*x*_Ni_*x*_S_2_, and CsCo_2–*x*_Ni_*x*_Se_2_

**DOI:** 10.1021/acs.chemmater.5c00996

**Published:** 2025-07-11

**Authors:** Ludmila Taskesen, Robert D. Smyth, Lemuel E. Crentsil, James I. Murrell, Emmanuelle Suard, Pascal Manuel, Simon J. Clarke

**Affiliations:** † Department of Chemistry, Inorganic Chemistry Laboratory, 6396 University of Oxford, South Parks Road, Oxford OX1 3QR, U.K.; ‡ Institut Laue-Langevin (ILL), BP 156, 71 Avenue des Martyrs, Grenoble 38042, France; § ISIS Pulsed Neutron and Muon Facility, Rutherford Appleton Laboratory, Harwell Oxford, Didcot OX1 10QX, U.K.

## Abstract

Layered transition metal chalcogenides are a versatile class of compounds that exhibit exotic physical phenomena, including superconductivity, thermoelectric properties and magnetic properties. The magnetic properties of ThCr_2_Si_2_-type solid solutions KCo_2–*x*_Ni_*x*_ *Ch*_2_ (*Ch* = S, Se; 0 ≤ *x* ≤ 2) with metallic properties were probed using magnetometry and powder neutron diffraction (PND). KCo_2_Se_2_ is ferromagnetic below ∼90 K and powder neutron diffraction (PND) showed evidence for long-range ferromagnetic order with localized moments of 0.6 μ_B_ per cobalt ion. With increasing nickel substitution, the system starts to order antiferromagnetically at *x* = 0.5. In these cases, PND experiments showed long-range A-type antiferromagnetic order with localized moments of around 1 μ_B_ per transition metal at 5 K. The Néel temperature (*T*_N_) for three-dimensional long-range ordering exhibits a maximum at *x* = 1, suggesting that nickel substitution enhances the antiferromagnetic interactions along the stacking direction. Higher nickel content suppresses the magnetic ordering temperature, and KCo_0.5_Ni_1.5_Se_2_ shows no magnetic long-range order with a lack of measurable Bragg peaks by PND (although a magnetic transition is evident by magnetometry), and further increasing the nickel content causes the system to become paramagnetic in the region 1.6 ≤ *x* ≤ 2. Our results show that increasing the electron count in the KCo_2–*x*_Ni_*x*_Se_2_ series has a dramatic effect on the physical properties. The analogous sulfide series - KCo_2–*x*_Ni_*x*_S_2_—shows similar behavior, and the series CsCo_2–*x*_Ni_*x*_Se_2_, containing a larger alkali metal ion, is comparable apart from the lack of a ferromagnetic region at high Co contents in the absence of an applied magnetic field.

## Graphical abstract

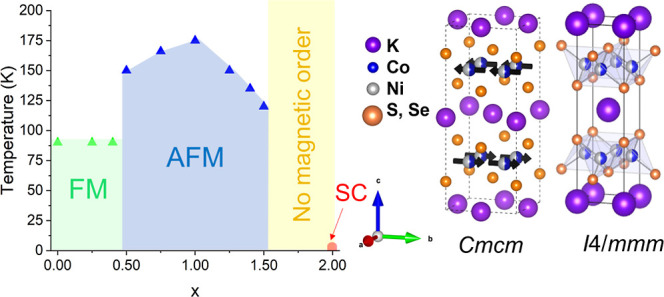

## Introduction

Layered transition metal compounds *AT*_2_ *X*_2_ (*A* = electropositive metal; *T* = transition metal; *X* = chalcogenide or pnictide) have been investigated in detail in recent years due to their versatile electronic and magnetic properties, including, but not confined to, unconventional superconductivity in the Fe-based pnictides and chalcogenides,^1^ metamagnetic behavior in Co-based selenides,^2^ and heavy-Fermion behavior in the Ni-based system KNi_2_Se_2_.^3^ These systems often crystallize in a versatile structure type often referred to as the ThCr_2_Si_2_-type.^4^ Here a number of chemical substitutions are possible and the associated properties are diverse, depending on structural details and electron count. The ThCr_2_Si_2_-type *AT*_2_ *X*_2_ structure (shown in Figure 1, and see ref 4) consists of covalently bonded transition metal chalcogenide *T*_2_ *X*_2_ layers stacked along the *c*-axis and separated by 8-coordinate electropositive *A* ions (*A* = alkali metal, alkaline earth metal, or lanthanide ion) which provide electrons.^2,4–6^ The transition metal, *T*, is coordinated tetrahedrally in *TX*_4_ units, and the shape of these edge-sharing tetrahedra determines the *T*–*T* distances and the details of the electronic structure, which in turn determines the electronic and magnetic properties. This gives rise to a sensitive dependence of properties on structure and composition.^4^ In the ThCr_2_Si_2_ structure there is *X*–*X* bonding parallel to the stacking direction between adjacent *T*_2_ *X*_2_ layers. In the chalcogenides described here there is no such bonding and to reflect this difference the structure type is often referred to as the BaZn_2_P_2_-type for such cases.^4^

**Figure 1:**
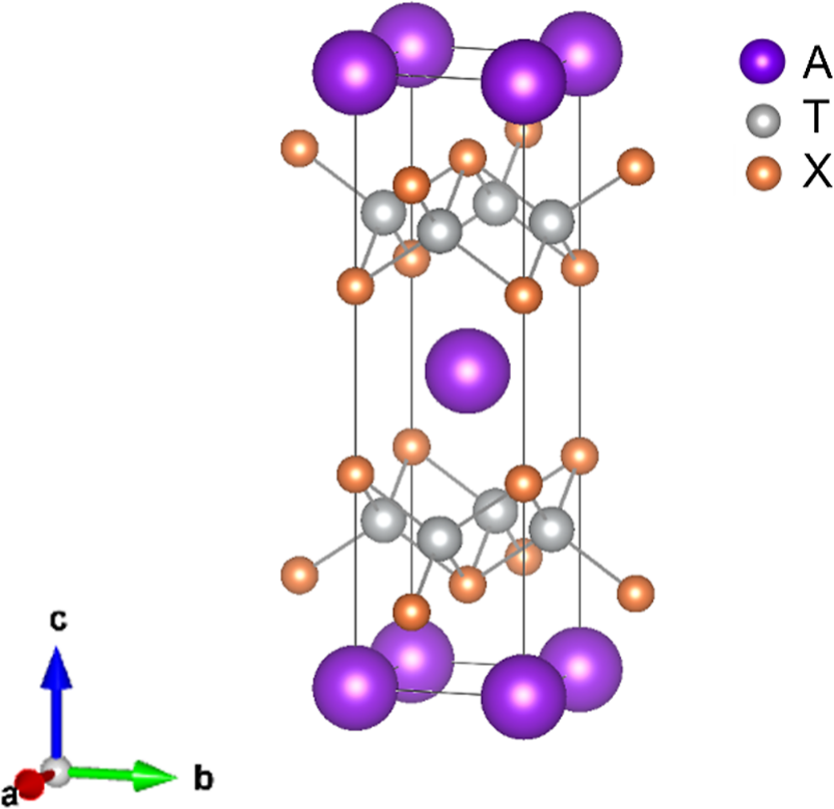
ThCr_2_Si_2_- and BaZn_2_P_2_-type structure *AT*_2_ *X*_2_ structure, where *A* = electropositive metal, *T* = d-block metal, and *X* = main-group anion-forming element.

For *AT*_2_ *X*_2_ compounds with *T* = Co in the chalcogenide layers,^2,6,7^ ferromagnetism within the layers is observed, but the between-plane ordering along the stacking axis is sensitive to composition and structural features. Ronneteg et al.^6^ reported on the TlCo_2_(Se_1–*x*_S_*x*_)_2_ solid solution, where selenium substitution for sulfide in ferromagnetic TlCo_2_S_2_ induces antiferromagnetism with a helical arrangement of the orientation of the ferromagnetic planes that is incommensurate along the stacking direction with a turning angle of 121° at 1.4 K, and the magnetic moment directions are therefore almost commensurate after three layers. While magnetometry shows that RbCo_2_Se_2_ and KCo_2_Se_2_ are ferromagnetic,^2,7^ in CsCo_2_Se_2_, single crystal neutron diffraction reveals A-type antiferromagnetic order below 100 K (i.e., in-plane ferromagnetism, but antiferromagnetism between planes),^8^ and there is a metamagnetic transition at higher fields that is not fully characterized.^2^ On the other hand, for *AT*_2_ *X*_2_ compounds with *T* = Ni in chalcogenide layers, and hence one additional electron per transition metal ion, Pauli paramagnetism is observed and superconductivity emerges at low temperatures.^3,9^ The structure can accommodate defects; for example in the Ni- and Fe-based systems, the *A* (electropositive metal) and *T* (transition metal) content was reported lower than stoichiometric, even when the 1:2:2 stoichiometry was targeted, namely K_0.64(4)_Fe_1.44(4)_Se_2_ ^10,11^ and K_0.95(1)_Ni_1.86(2)_Se_2_.^12^ In the Fe systems, the highly deficient phases have Fe/vacancy order^10^ and are antiferromagnetic and insulating but they sometimes coexist with *A*_*x*_Fe_2_Se_2_ compositions which are metallic and show high-temperature superconductivity.^13^ With *A* = Tl, Ni/Co mixing in the *T*_2_ *X*_2_ transition metal chalcogenide layers^14,15^ results in A-type antiferromagnetism: ferromagnetic coupling within the plane as the result of direct magnetic interactions between the metal moments coexists with antiferromagnetic ordering along the stacking (*c*) axis, which is assumed to operate via the Ruderman-Kittel-Kasuya-Yosida (RKKY) mechanism.^6,14,15^

In this work we investigate the change in magnetic properties of the solid solution between KCo_2_Se_2_ and KNi_2_Se_2_ and the sulfide and Cs analogues. The end members crystallize in the BaZn_2_P_2_ variant of the ThCr_2_Si_2_-type structure (i.e., no chalcogen–chalcogen bonding) but differ in terms of their magnetic properties. Upon substitution of Co in KCo_2_Se_2_ by Ni, an electron is added to the 3d bands per Ni ion, which drastically alters the magnetic properties across the whole series. Using powder neutron diffraction (PND) and magnetometry, we determine the magnetic regions of the solid solutions KCo_2–*x*_Ni_*x*_Se_2_, KCo_2–*x*_Ni_*x*_S_2_, and CsCo_2–*x*_Ni_*x*_Se_2_, and contrast the magnetic behavior to related mixed-transition-metal chalcogenides with similar structures.

## Experimental Section

### Synthesis

#### Synthesis of KCo_2–*x*_Ni_*x*_Se_2_

Samples of KCo_2–*x*_Ni_*x*_Se_2_ (0 ≤ *x* ≤ 2) were prepared with 0.25 increments in *x* from stoichiometric amounts of selenium (99.99%, Alfa Aesar), nickel (99.99%, Alfa Aesar) and, cobalt powders (99.99%, Alfa Aesar) and potassium selenide (K_2_Se) powder prepared in-house (see below). The mixtures were ground, placed in alumina crucibles covered with loose-fitting alumina lids and flame-sealed in evacuated silica tubes under vacuum (∼10^–2^ mbar) with the end of the tube submerged in liquid nitrogen during the sealing process to prevent vaporization of elemental selenium. The sealed tubes were placed in a furnace for a pretreatment, to enable the relatively volatile chalcogen to react, ensuring the vapor pressure within the sealed ampules did not become too great, which involved slow heating at a rate of 1 °C min^–1^ to 400 °C followed by a 4 h dwell and then cooling at the natural rate of the furnace. The powders were then reground and reheated to 700 °C for 72 h, followed by slow cooling at a rate of 0.1 °C min^–1^ to room temperature. Products were dark red-brown polycrystalline powders. It is important to note that KNi_2_Se_2_ could not be synthesized phase pure via the above-mentioned route, as competing impurities, including KNi_3_Se_3_, NiSe and Ni_3_Se_2_, were present in the PXRD patterns. Another synthesis route was employed in this case (see below).

#### Synthesis of KCo_2–*x*_Ni_*x*_S_2_

Samples of KCo_2–*x*_Ni_*x*_S_2_ with 0 ≤ *x* ≤ 1.75 and 0.25 increments in *x* were prepared using the same method described for the selenide analogue above, using K_2_S prepared in-house (synthesis described below).

#### Synthesis of CsCo_2–*x*_Ni_*x*_Se_2_

Samples of CsCo_2–*x*_Ni_*x*_Se_2_ with 0.25 ≤ *x* ≤ 1.75 were synthesized from elemental Cs (99.98%, Alfa Aesar), and previously prepared nickel selenide (NiSe) and cobalt selenide (CoSe). These precursors of composition NiSe and CoSe were made by heating pressed pellets of the elements at 900 °C (NiSe) or 600 °C (CoSe) for 12 h in evacuated silica tubes. To synthesize the ternary phases, a stoichiometric amount of Cs was placed in an alumina crucible with a loose-fitting lid with the powdered NiSe and CoSe precursors and sealed in evacuated silica tubes under vacuum (∼10^–2^ mbar) with the end of the tube submerged in liquid nitrogen to prevent vaporization of elemental cesium during the sealing process. The sealed tubes were slowly heated to 450 °C at a rate of 1 °C min^–1^ and the temperature was maintained for 6 h, followed by furnace cooling. The powders were reground and reheated to 750 °C for 72 h, followed by quenching the tubes in an ice bath to room temperature. Products were dark brown polycrystalline powders.

### Preparation of Precursors

K_2_Se and K_2_S precursors were prepared from K ingot (99.95%, Alfa Aesar) and Se/S powder on a Schlenk line. The elements were placed in a Schlenk tube and ammonia was condensed onto them at −78 °C in a dry ice/isopropanol bath and this temperature was maintained for ∼3 h while stirring on a stirrer plate. Once reaction was complete at low temperatures (marked by color change from blue solution to orange suspensions), ammonia was allowed to evaporate by allowing the solution to warm slowly to room temperature by removing the dry ice/isopropanol bath. After the solid residue had reached room temperature the Schlenk tube was evacuated to remove any adsorbed NH_3_. Orange powders were the products of the reactions and their purity was confirmed by PXRD, where no other phases were detected. (Caution: ammonia is volatile and toxic. The synthesis was performed in a fume hood and at all times the liquid-ammonia-containing vessel was open to a mercury bubbler to avoid the possibility of pressures exceeding 50 mmHg above atmospheric pressure in the reaction vessel).

### Synthesis of KNi_2_Se_2_ and KNi_2_S_2_

Single crystal growth using a so-called “self-flux” method was employed to obtain high purity samples of these compounds. Stoichiometric amounts of K ingot and NiSe or NiS powder precursors were placed in an alumina crucible, covered with an alumina lid, and sealed in an evacuated silica tube under vacuum (∼10^–2^ mbar) with the end of the tube submerged in liquid nitrogen to prevent vaporization of elemental K during the sealing process. The sealed tubes were placed in a furnace and heated slowly to 1000 °C at a rate of 1 °C min^–1^, held at 1000 °C for 3 h, and then slowly cooled to 700 °C at 0.1 °C min^–1^, after which the furnace was shut off and allowed to cool at its natural rate over a few hours. Bronze-colored crystals were obtained (typically 0.2 × 0.2 × 0.3 mm in size). A small amount of NiSe impurity was detected by PXRD in the ground bulk product in the case of the selenide.

### Powder Diffraction Measurements

Laboratory powder X-ray diffraction (PXRD) instruments were used to follow synthetic progress (Bruker D8 Advance Eco, CuKα_1_/Kα_2_, and Malvern PANalytical Empyrean Alpha1 (CuKα_1_ only, selected using a Ge crystal (111) monochromator)). High-resolution diffraction patterns were collected using the instrument I11 at the Diamond Light Source Ltd., UK with 0.826 Å X-rays (calibrated using a Si standard before each series of experiments)^16^ at room temperature and at 100 K using the multianalyzer crystal (MAC) detector of this instrument. Variable temperature PXRD patterns were obtained every 24 s using the MYTHEN position sensitive detector (PSD) on cooling in a nitrogen cryostream between 300 and 100 K at a rate of 6 K min^–1^. Powder neutron diffraction (PND) data were collected on the D2B instrument at the Institut Laue Langevin (ILL), Grenoble, France^17^ (λ = 1.594 Å selected using a Germanium [115] crystal as monochromator), or on the WISH diffractometer at the ISIS pulsed neutron and muon source, Harwell, UK.^18^ The contrasting scattering lengths of Co (2.50 fm) and Ni (10.3 fm) mean that these measurements are sensitive to composition and/or Co/Ni order as well as magnetic long-range order. For these experiments approximately 2 g of each material were loaded into 6 mm diameter vanadium cans sealed with indium wire gaskets which provided a hermetic seal and the neutron diffraction data were obtained at various temperatures: between 3.5 and 300 K using a closed-cycle refrigerator on D2B and between 1.5 and 295 K on WISH using an Oxford Instruments cryostat. For each sample, analysis of any magnetic contribution to the PND data was carried out using the ISODISTORT software^19^ to generate possible magnetic irreducible representations. Rietveld refinements against PXRD and PND data used the TOPAS Academic V6 software^20^ to refine the magnetic and nuclear models to fully account for the Bragg peak intensities.

### Magnetometry

The magnetic moments of samples of each compound were measured using a Quantum Design MPMS3 magnetometer. For each measurement, 10–30 mg of powder was weighed accurately into a gelatin capsule which was fastened into a plastic straw and secured inside the magnetometer. The possible presence of a ferromagnetic impurity was first determined by measuring the magnetic moment of the sample (proportional to the magnetization (*M*)) against applied magnetic field (*H*). Linear relationships between the two, even at low fields (<1000 Oe), signaled the lack of ferromagnetic impurities. Magnetic susceptibility was determined by measuring the magnetic moment of the sample as a function of temperature on warming from 2 to 300 K after cooling both in a zero applied field (zero-field-cooled - ZFC) and in the measuring field (field-cooled - FC) of 1000 Oe. Magnetisation isotherms (−7 ≤ μ_0_ *H*/T ≤ 7) were measured at room temperature and at low temperature (2 K) after cooling the sample from 300 K to the measurement temperature in a 5 T field.

### SEM–EDX

SEM measurements were carried out in the David Cockayne center for Electron Microscopy, University of Oxford using a ZEISS EVO MA10 microscope with an operating voltage of 20 kV and probe current of 4.0 nA equipped with an Oxford Instruments X-act energy-dispersive X-ray (EDX) analysis system with Aztec software to enable semiquantitative chemical analysis to establish compositions and homogeneity. A small amount of powder sample was sprinkled on adhesive carbon tape mounted on a sample holder for insertion into the SEM instrument. This was done under inert atmosphere within the glovebox and the samples were sealed in a polyethylene bag for transport to the SEM instrument so that there was minimal air exposure between mounting the samples and loading them in the SEM. Mapping measurements for particle homogeneity were carried out and compositions were estimated by averaging point measurements over 3–5 sites per particle for each of several particles. We also probed a sample of stoichiometric K_2_Se (made in-house on the Schlenk line) to account for systematic overestimation of Se content by the software and this produced a measured atomic ratio of K/Se of 1.86:1. We corrected the measurements of the ternary phases for this overestimation of the Se content.

### Single Crystal X-ray Diffraction

Single crystal X-ray diffraction data were collected using a Rigaku Oxford Diffraction Supernova-A diffractometer using Mo-*K*α (λ = 0.71073 Å) radiation. Single bronze-colored plate-shaped crystals were handled under argon using a Schlenk line, enabling them to be dispersed in oil under anaerobic conditions. Suitable crystals were selected and mounted on the instrument on a MiTeGen loop and rapidly cooled to 150 K using an Oxford CryoSystems CryoStream 700 Plus. Data collection and reduction were performed using the CrysAlis^PRO^ software.^21^ A preliminary experiment was conducted for each crystal to assess the quality of diffraction (based on the intensity and shape of the peaks) and to determine the metric symmetry of the crystal. For an apparent single crystal with nominal composition “KNi_2_Se_2_” it was evident that a NiSe impurity was present as a second crystal, but the remaining peaks were indexed on a tetragonal unit cell with cell parameters matching those for the KNi_2_Se_2_ refined from powder X-ray diffraction measurements on the bulk. The integrated intensities of the reflections were then corrected for Lorentz-polarization and absorption using the multiscan absorption correction within CrysAlis^PRO^. In all, 1504 reflections were merged into 121 independent ones with *R*_int_ = 0.123, of which 103 reflections satisfied the criterion *I* > 3σ­(*I*). The structure was solved by charge-flipping using SuperFlip^22^ and refined by full-matrix least-squares refinement using the Jana2020 software.^23^ Anisotropic displacement parameters were refined for all atoms as well as the occupancy of K. The refined composition of the crystal from this analysis is presented in Table S3 and compared with literature values.^12^ However, the crystal was of relatively low quality resulting in significant residual electron density and other samples did not produce crystals of sufficiently size or quality for this measurement.

## Results and Discussion

### KCo_2–x_Ni_*x*_Se_2_ Series

#### Crystal Structure

The entire solid solution KCo_2–*x*_Ni_*x*_Se_2_ with 0 ≤ *x* ≤ 2 was synthesized with good purity (>95%) according to Synchrotron PXRD. Crystalline secondary phases present are small amounts of either NiSe or CoSe, depending on composition. PND revealed, from its magnetic scattering, a CoO impurity (<5%), which was barely detected by PXRD (CoO has a large localized magnetic moment on Co,^24^ and the magnetic scattering is mostly concentrated in a single intense reflection). PXRD patterns of all 7 measured samples in the KCo_2–*x*_Ni_*x*_Se_2_ series (Figure 2), were indexed according to the tetragonal ThCr_2_Si_2_-type structure (Space group: *I*4/*mmm*, no.139) of the parent stoichiometric phases using Rietveld refinement, indicating the formation of a solid solution across the whole 0 ≤ *x* ≤ 2 range. Rietveld refinements of room temperature Synchrotron PXRD and PND patterns of KCoNiSe_2_ (middle member of the series) are shown in Figure 3a,b, respectively, together with the crystal structure obtained from the Rietveld refinement (Figure 3c and Table S1). The variations of the unit cell parameters *a* and *c*, unit cell volume, and the *c*/*a* ratio with *x* in KCo_2–*x*_Ni_*x*_Se_2_ are shown in Figure 4. For pure KCo_2_Se_2_, *a* = 3.841(3) Å, and *c* = 13.8020(2) Å, which is in good agreement with previous work on this phase.^2^ As the Ni content increases from *x* = 0 to 2 in KCo_2–*x*_Ni_*x*_Se_2_, the basal lattice parameter *a* increases by 1.6%, while there is a more pronounced decrease in the *c* parameter, which shrinks by 2.6%. Overall, the unit cell volume shows a general increase with increasing Ni content. The trends agree with the work of Newmark et al.^14^ on the related solid solution TlCo_2–*x*_Ni_*x*_Se_2_. The interatomic distances and tetrahedral angles across the series are shown in Figure S1. All unit cell parameters, interatomic distances and angles are shown in Table S2 for the KCo_2–*x*_Ni_*x*_Se_2_ series.

**Figure 2:**
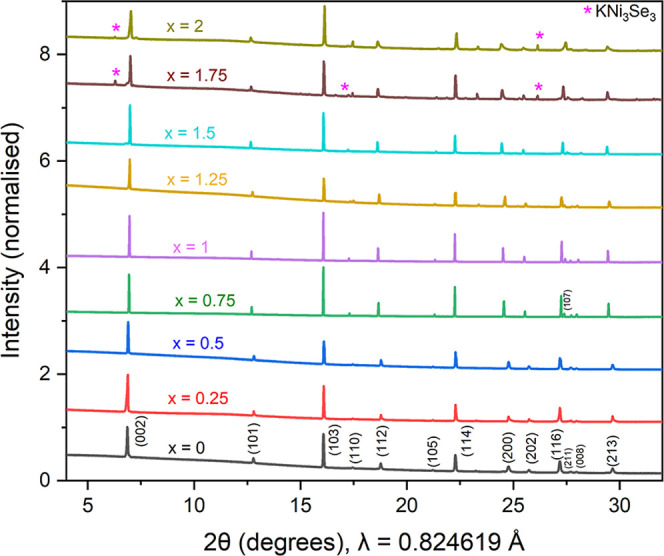
Synchrotron PXRD patterns of KCo_2–*x*_Ni_*x*_Se_2_ with 0 ≤ *x* ≤ 2. The impurity marked by a magenta asterisk in *x* = 1.75 and 2 is KNi_3_Se_3_.

**Figure 3:**
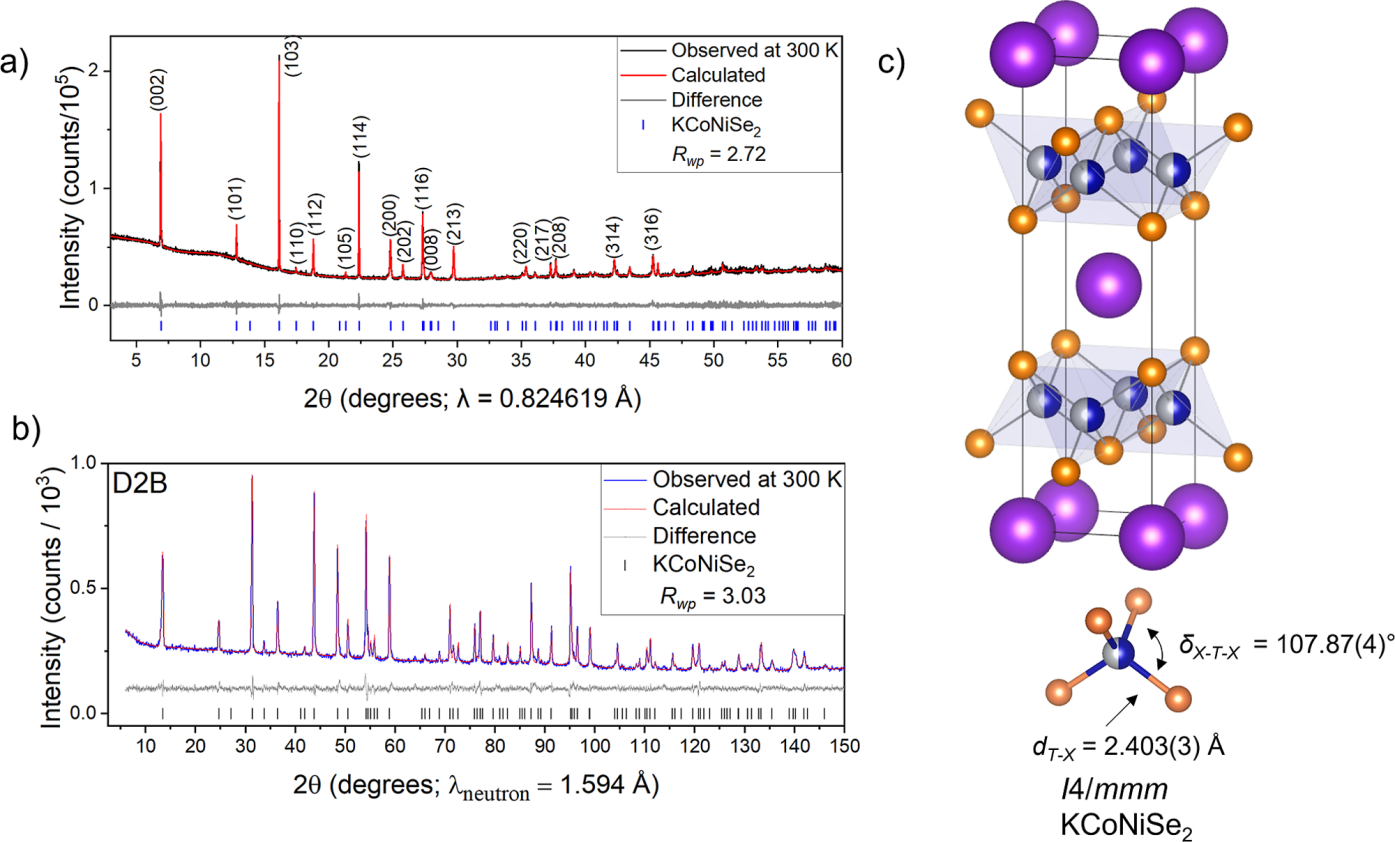
Room-temperature Rietveld refinement of KNiCoSe_2_ against (a) synchrotron PXRD and (b) PND (D2B diffractometer, ILL, Grenoble). (c) Crystal structure of KNiCoSe_2_ obtained from the Rietveld refinement against PND data. The Co/NiSe_4_ tetrahedron is distorted by slight compression along the *c* axis (the angle shown which has a multiplicity of 4 has a value of 107.87°, less than the ideal angle of 109.47°).

**Figure 4:**
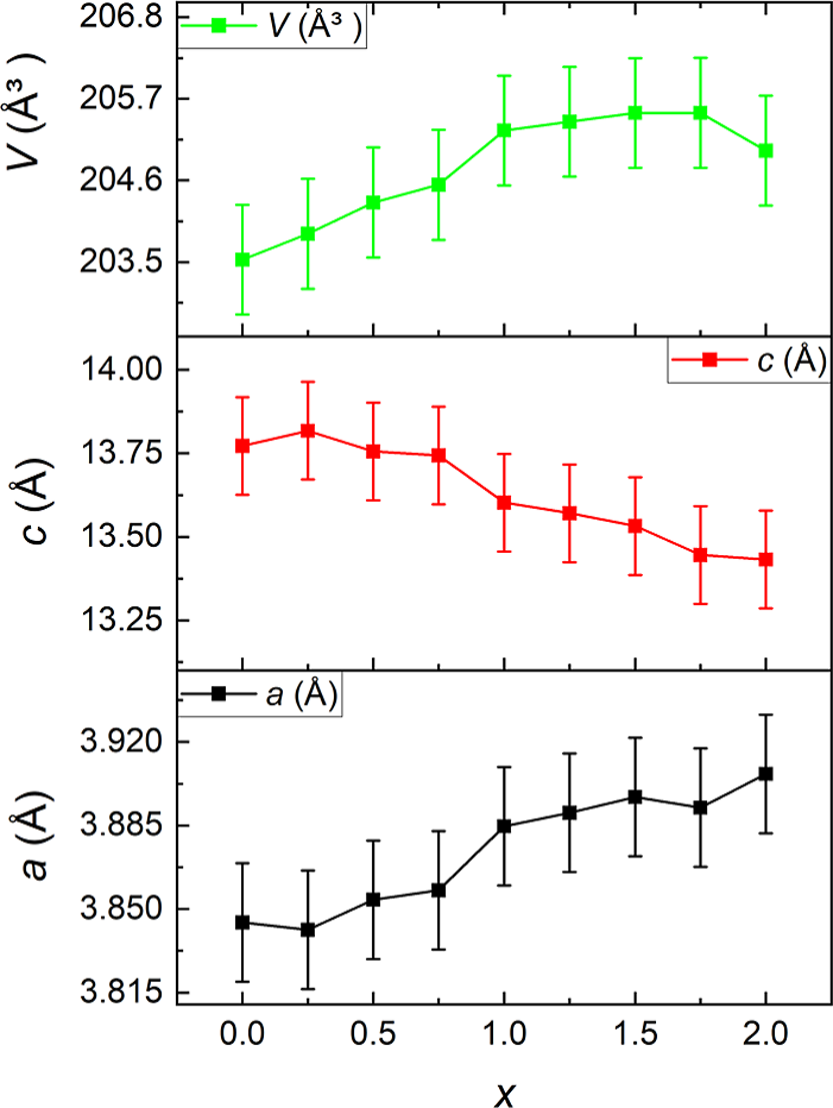
Change in lattice parameters *a*, *c*, and unit cell volume (*V*) with *x* in the KCo_2–*x*_Ni_*x*_Se_2_ series obtained from PXRD measurements using the MAC detector at I11 (Diamond).

### Compositional Analysis

Previous literature on KNi_2_Se_2_ single crystals and other Ni-containing ThCr_2_Si_2_-type compounds reports nonstoichiometry on both K and Ni sites. Lei et al.^12^ report on single crystals of K_*x*_Ni_2–*y*_Se_2_ with composition *x* = 0.95(1), *y* = 0.14(2) derived from single crystal XRD refinement and EDX analysis. Similar Ni-containing systems, including LaNi_1.88_P_1.9_,^25^ EuNi_1.88_As_1.92_,^26^ are also reported as nonstoichiometric, and a homogeneity range was established for SrNi_*x*_Sb_2_ with 1.72 < *x* < 1.85.^27^ It was proposed that the Ni deficiency could be caused by the necessity to stabilize the electronic structure by removing an excess of valence electrons.^28^ Similar nonstoichiometry is also reported in Co-containing ThCr_2_Si_2_-type pnictides, including ACo_2–*x*_As_2_ (A = Ca, La, Ce, Pr, Nd).^29,30^ Our Single Crystal X-ray diffraction (SC XRD) data show a similar trend: the single crystal with nominal composition KNi_2_Se_2_ was ∼10% deficient on the potassium site with a refined composition of K_0.90(3)_Ni_1.948(14)_Se_2.00(9)_ (see Table S3). This result and the structural parameters agree relatively well (within 3 × the estimated standard deviations on the refined parameters) with the work of Lei et al.,^12^ who reported the phase as K_0.95(1)_Ni_1.86(2)_Se_2.00(1)_. The SEM–EDX data collected on KNi_2_Se_2_ is in good agreement with the SC XRD data on the same sample; the composition from EDX is K_0.90(6)_Ni_2.01(9)_Se_2.00(9)._ Attempted single crystal X-ray analysis of a candidate single crystal of KCoNiSe_2_ as a representative of the KCo_2–*x*_Ni_*x*_Se_2_ series showed severely elongated spots (Figure S2), suggesting high mosaicity, possibly due to compositional inhomogeneity or stacking faults. We then relied on SEM–EDX analysis to back-up the powder diffraction measurements in the analysis of the composition of the nonstoichiometric polycrystalline members of the series. Elemental analysis by SEM–EDX reveals an even distribution of K, Co, Ni, and Se across a portion of the polycrystalline sample of KCoNiSe_2_ (Figure 5). Figure S3 shows an SEM image of a second representative of KCoNiSe_2_, where there are visible potassium-rich regions that do not contain any other element, including oxygen, and these are of uncertain origin. Table 1 summarizes the data collected from SEM–EDX analysis on compositions KCo_2–*x*_Ni_*x*_Se_2_ and compares them with the refined atomic occupancies from PND analysis. The atomic ratios obtained from SEM–EDX obtained by scaling against the K_2_Se standard as described in the experimental section are shown in Table 1 (Ratios obtained prior to scaling from the K_2_Se standard can be found in Table S4). These values carry quite large estimated standard deviations on the element content, but suggest slight K-deficiency, but no deficiency on the transition metal sites. For KCoNiSe_2_ there was a slight K deficiency according to EDX (even after calibrating for the apparent Se excess in the measured data)—observed composition of K_0.89(3)_Co_1.18(6)_Ni_1.02(6)_Se_2.00(1)_ and PND analysis (K_0.92(1)_Co_0.98(1)_Ni_1.02(1)_Se_2.00(1)_), although the compositions are similar considering the uncertainties. KCo_2_Se_2_ also showed a K-deficient composition in EDX—K_0.88(6)_Co_2.10(7)_Se_2.00(1)_, while the composition from PND analysis is slightly Co-deficient (K_1.00(2)_Co_1.92(3)_Se_2.00(1)_) (although the uncertainty is relatively high because of the short scattering length of Co). The Co/Ni ratio was determined from PND rather than XRD, as the two metals have very different neutron scattering lengths. (Co: 2.50 fm; Ni: 10.3 fm). The refined Co/Ni ratios were observed to be in good agreement with nominal compositions in the analysis of both PND and SEM–EDX data.

**Figure 5:**
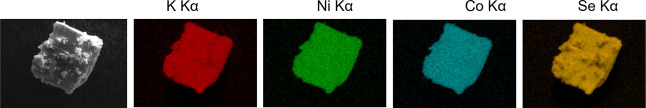
An image of a polycrystalline sample of KCoNiSe_2_ sample under SEM, showing an even distribution of the constituent elements.

**Table 1:** Nominal and Observed Compositions from SEM–EDX and PND Data on KCo_2–*x*_Ni_*x*_Se_2_

nominal composition	observed composition from SEM–EDX	observed composition from PND
KCo_2_Se_2_	K_0.88(6)_Co_2.10(7)_Se_2.00(1)_ ^a^	K_1.00(2)_Co_1.92(3)_Se_2.00(1)_ ^a^
KCo_1.75_Ni_0.25_Se_2_	K_0.86(2)_Co_1.74(7)_Ni_0.19(2)_Se_2.00(1)_	
KCo_1.5_Ni_0.5_Se_2_	K_0.89(3)_Co_1.47(8)_Ni_0.47(3)_Se_2.00(1)_	K_0.95(1)_Co_1.44(1)_Ni_0.56(1)_Se_2.00(1)_
KCo_1.25_Ni_0.75_Se_2_	K_0.89(2)_Co_1.24(1)_Ni_0.79(5)_Se_2.00(1)_	
KCoNiSe_2_	K_0.89(3)_Co_1.18(6)_Ni_1.02(6)_Se_2.00(1)_	K_0.92(1)_Co_0.98(1)_Ni_1.02(1)_Se_2.00(1)_
KCo_0.75_Ni_1.25_Se_2_	K_0.98(2)_Co_0.83(6)_Ni_1.23(7)_Se_2.00(1)_	
KCo_0.5_Ni_1.5_Se_2_	K_1.00(4)_Co_0.45(4)_Ni_1.46(1)_Se_2.00(2)_	K_1.00(1)_Co_0.44(1)_Ni_1.56(3)_Se_2.00(1)_
KNi_2_Se_2_	K_0.90(6)_Ni_2.01(9)_Se_2.00(9)_	

a The standard deviations in the EDX measurement come from multiple independent measurements over the sample. The estimated standard deviations from the PND experiments are estimated from the Rietveld software based on data quality and parameter correlations.

### Resistivity Measurements

The resistivity (ρ) measurement on a representative sample of KCoNiSe_2_ was performed on a small piece of a sintered pellet connected to four copper wires using silver paint and mounted on a custom-made probe. The resistivity of KCoNiSe_2_ increases linearly with increasing temperature, corresponding to metallic behavior in the temperature range 30–300 K, with ρ of the order of 10^–2^ Ω cm at room temperature (Figure 6). Further quantitative comparisons of the resistivity of the series would require larger single crystal samples to eliminate grain boundary effects.

**Figure 6:**
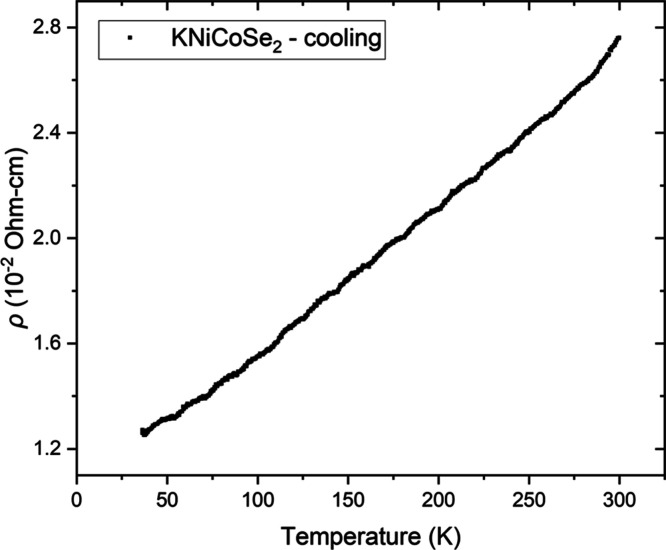
Resistivity as a function of temperature for KNiCoSe_2_ measured on cooling on a pressed pellet of material. The slight wiggling of the data is attributed to measurement noise.

### Magnetic Properties of KCo_2–*x*_Ni_*x*_Se_2_

As the end members of the solid solutions have differing magnetic behaviors (KCo_2_Se_2_ is a ferromagnetic metal,^2^ and KNi_2_Se_2_ is a paramagnetic metal with a low temperature superconducting transition^3^), mapping out the changes in magnetic behavior of KCo_2–*x*_Ni_*x*_Se_2_ solid solution was carried out using magnetometry and PND on selected compositions. Figure 7 shows the magnetic susceptibility, χ, as a function of temperature for the seven nonstoichiometric samples of the solid solution. At high cobalt contents in the KCo_2–*x*_Ni_*x*_Se_2_ series (0 ≤ *x* ≤ 0.25), ferromagnetic behavior is observed with *T*_C_ ∼ 90 K in the KCo_2_Se_2_ end member,^2^ diminishing to about 65 K for *x* = 0.25 (low temperature magnetization isotherms for the two ferromagnetic members—*x* = 0 and 0.25—are shown in Figure S4). KCo_2–*x*_Ni_*x*_Se_2_ for 0.5 ≤ *x* ≤ 1.5 show antiferromagnetic behavior. The Néel temperatures, *T*_N_, as judged by the temperature of the susceptibility maximum, initially increase with increasing *x*, reach a maximum value of 175 K at *x* = 1, and then decrease with increasing *x*. At high nickel contents: 1.5 < *x* ≤ 2, paramagnetic behavior is observed down to low temperatures with evidence of a magnetic transition of uncertain origin around 10 K. Figure 8 depicts a magnetic phase diagram for KCo_2–*x*_Ni_*x*_Se_2_ constructed from magnetometry analysis, and neutron diffraction, summarizing the four magnetic regions in the solid solution. The attempted Curie–Weiss fits for *x* = 1, 1.75, and 2 can be found in Figures S5, S6 and Table S5 summarizes the resulting parameters obtained from the fitting. While the data show linear dependence of the inverse susceptibility against temperature in the region just below room temperature, the fact that the highest measurement temperature is not much greater than the temperatures attributable to the long-range magnetic ordering from neutron diffraction (see below) mean that attempts to interpret the magnetometry using the Curie–Weiss model should be treated with caution (see Supporting Information, Table S5).

**Figure 7:**
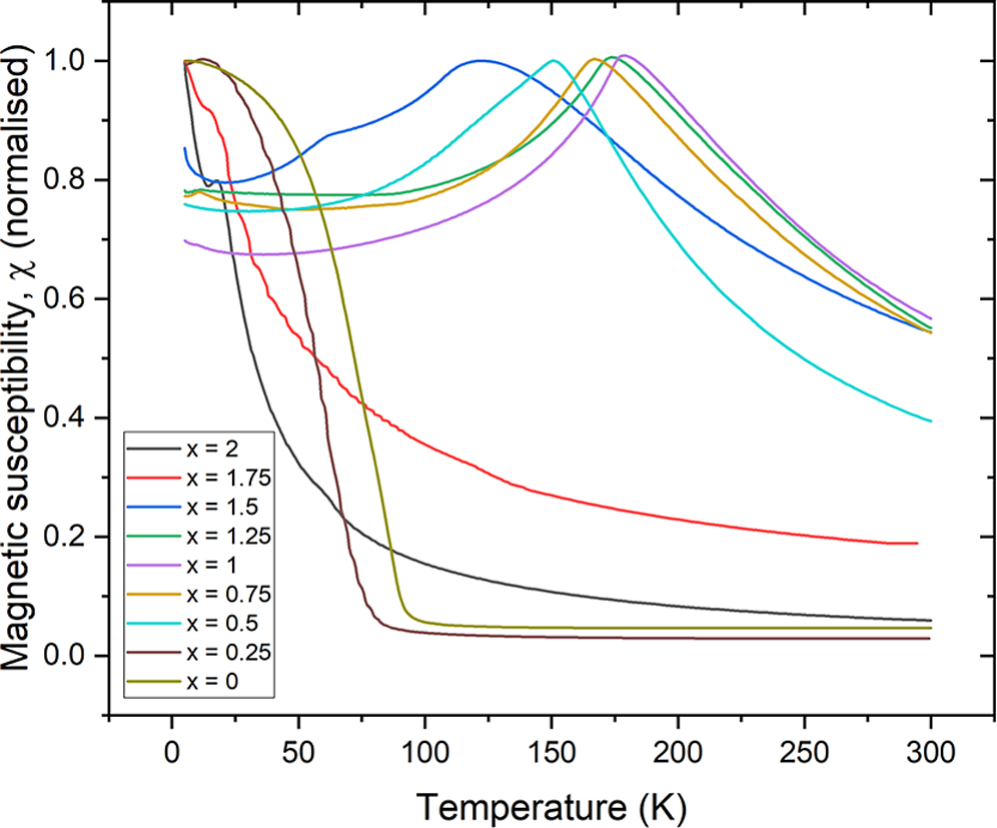
Magnetic susceptibility (ZFC measurements) as a function of temperature for the series KCo_2–*x*_Ni_*x*_Se_2_, 0 ≤ *x* ≤ 2. The susceptibility values have been normalized to emphasize the transitions. The ferromagnetic samples have susceptibilities about 100 times greater than the antiferromagnetic samples (see Figure S20).

**Figure 8:**
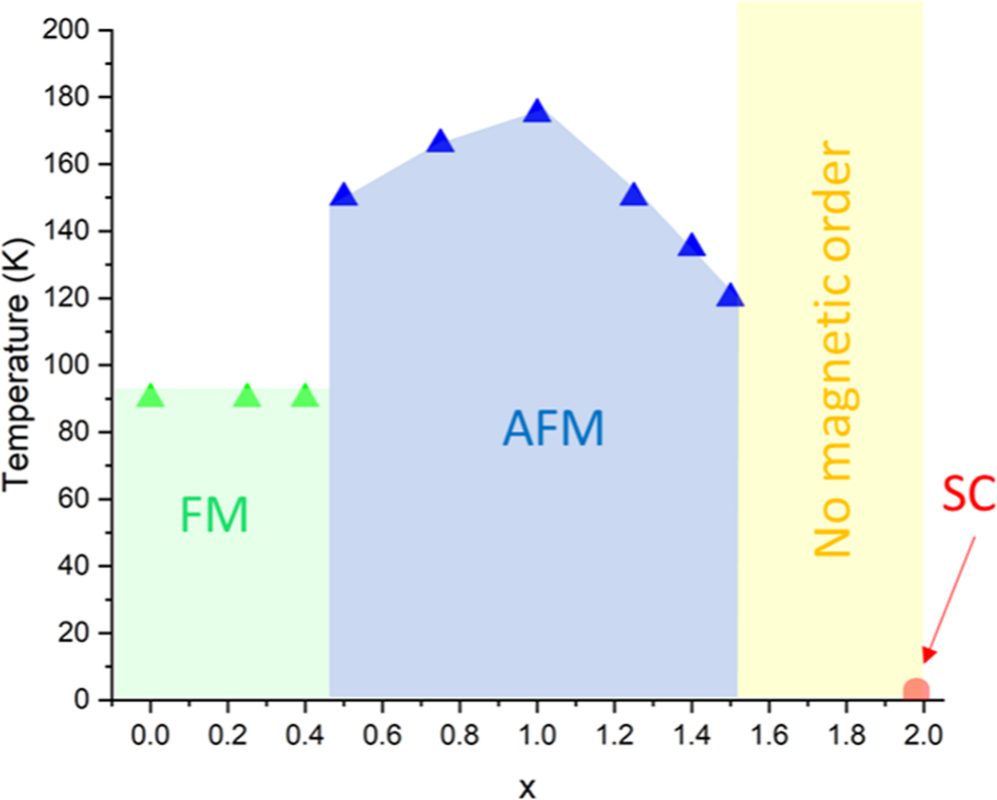
Magnetic phase diagram for KCo_2–*x*_Ni_*x*_Se_2_ (0 ≤ *x* ≤ 2) showing the ferromagnetic region (green), antiferromagnetic region (blue), paramagnetic region (yellow), and superconducting region (red).

### Magnetic Ordering of KCo_2–*x*_Ni_*x*_Se_2_

Three antiferromagnetic members of the series, KCo_0.5_Ni_1.5_Se_2_, KCoNiSe_2_, and KCo_1.5_Ni_0.5_Se_2_, were measured using PND to probe long-range magnetic order. Low temperature (3.5 K) PND measurements on KCoNiSe_2_ (Figure 9a) and KCo_1.5_Ni_0.5_Se_2_ (Figure 9b) revealed low angle (001), (003), and (005) Bragg reflections which are systematically absent for the nuclear structure, so must be of magnetic origin. In the Ni-rich phase KCo_0.5_Ni_1.5_Se_2_ these reflections were not evident (Figure S7). The magnetic scattering of KCoNiSe_2_ and KCo_1.5_Ni_0.5_Se_2_ at 3.5 K was indexed using a √2*a* × √2*a* × *c* expansion of the nuclear unit cell ((1/2 1/2 0) propagation vector). Initially no symmetry constraints were applied to the magnetic structure, but it was found that a single magnetic mode, mM5–, was found to account for all magnetic peaks of the structure at 2θ = 6.75° (001), 20.07° (003), 34.63° (005), and 39.95° (203) (hkl values refer to the expanded cell), which corresponds to ferromagnetic coupling between Co/Ni ions within the (Co/Ni)_2_Se_2_ sheets and an antiferromagnetic interaction between the adjacent (Co/Ni)_2_Se_2_ sheets along the stacking axis. The magnetic-plus-nuclear structure can be described with the orthorhombic space group *Cmcm* (63.468 in the Belov–Neronova–Smirnova (BNS) scheme^31^), as opposed to the tetragonal symmetry (*I*4/*mmm*) of the nuclear-only structure of the KCo_2–*x*_Ni_*x*_Se_2_ series. In order to investigate whether a reduction in symmetry of the nuclear structure below the magnetic ordering transition was observable, we measured high-resolution synchrotron PXRD patterns of KCoNiSe_2_ at 300 and 100 K and the 200 peak of the *I*4/*mmm* structural cell was fitted with a Gaussian peak shape with a fwhm (full width at half-maximum) of 0.09951° and 0.09202° extracted at 100 and 300 K, respectively, resulting in a broadening of 0.00749° at low temperatures (Figure S8a,b). The peak broadening of other peaks, namely 112 (Figure S8c,d) and 213 (Figure S8e,f), was calculated to be 0.00558° and 0.0156° at 100 K, respectively. However, the X-ray data are best fit with a tetragonal structural model, and there is no clear evidence for a structural distortion even using a high-resolution diffractometer, although the arrangement of magnetic moments is not tetragonal and needs to be described by the orthorhombic model. In the refinements in Figure 9a,b we refined a nuclear model in *I*4/*mmm* and a separate magnetic model containing just the transition metal moments with symmetry *Cmcm*, but with a metrically tetragonal √2*a* × √2*a* × *c* expansion of the nuclear unit cell. Thus, separate tick-marks show the reflections from the nuclear model and the magnetic model in Figure 9. The refined magnetic structure is A-type antiferromagnetic with ferromagnetic coupling of Co/Ni moments within the *ab* plane and the planes coupled antiferromagnetically along the *c* axis (Figure 9c). The likely mechanism for ferromagnetic coupling in the *ab* plane is direct exchange as *d*_*T*‑*T*_ is 2.74 Å, which is comparable to that observed in the elemental metals (*d*_Co–Co_ = 2.51 Å in Co metal). The interlayer *T*–*T* distance is too large for any direct interaction. Antiferromagnetic coupling between planes has been proposed to operate via the RKKY mechanism via conduction electrons in this series of metallic compounds,^4,6,8,14,32^ which is sensitive to the nature of the interlayer electropositive ion,^33^ the nature of the band structure, influenced strongly by the electron count, and the interlayer separation. The refined long-range ordered moment of Co/Ni ions in KCoNiSe_2_ is 1.18 μ_B_, while in KCo_1.5_Ni_0.5_Se_2_ it is 1.07 μ_B_, the small localized moments are consistent with other electrons being mobile conduction electrons giving rise to metallic behavior (Figure 6). As no superstructure peaks were observed from PND data above the magnetic transition, the nickel and cobalt ions, with very different neutron scattering lengths, must be disordered in the (Co/Ni)_2_Se_2_ layers. PND patterns measured at room temperature of these phases are shown in Figures S9–S12.

**Figure 9:**
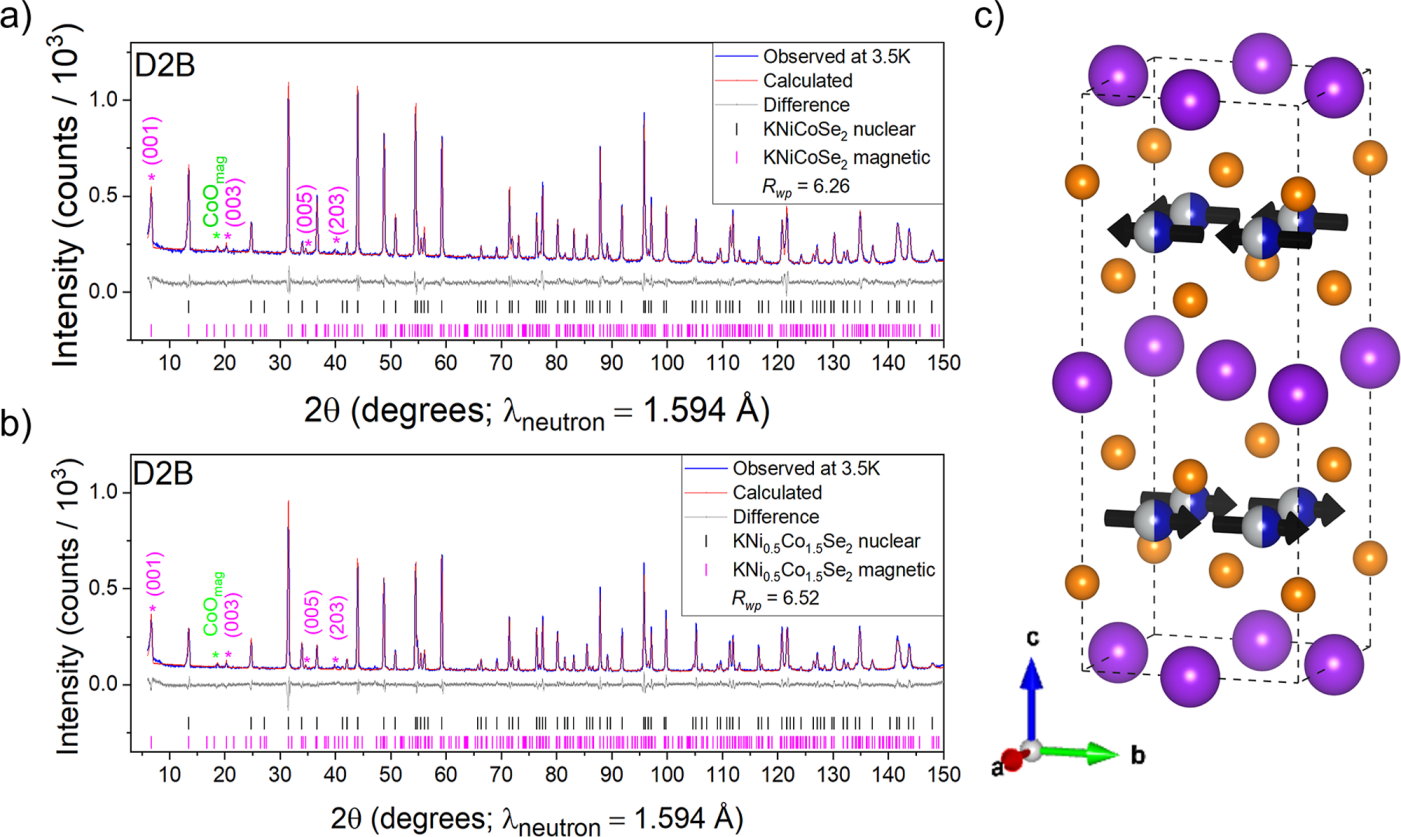
(a) Rietveld refinement of KCoNiSe_2_ against PND data measured at 3.5 K, *R*_wp_ = 6.26% and χ^2^ = 1.83. (b) Rietveld refinement of KCo_1.5_Ni_0.5_Se_2_ against PND data measured at 3.5 K, *R*_wp_ = 6.52% and χ^2^ = 2.08. (c) Magnetic plus nuclear structure for KCoNiSe_2_ and KCo_1.5_Ni_0.5_Se_2_, with ferromagnetic coupling of Co/Ni moments in the *ab* plane and antiferromagnetic coupling of Co/Ni moments along the stacking axis. Purple asterisks mark magnetic reflections which are not present at room temperature.

A variable temperature PXRD measurement was carried out on KCoNiSe_2_ between 100 and 300 K to monitor the changes in lattice parameters and unit cell volume (Figure 10a). While the volume decreases linearly on cooling in this range, nonlinear trends were observed for the *a* and *c* lattice parameters and for the *c*/*a* ratio which increases sharply at the *T*_N_ of 175 K deduced from the magnetic susceptibility measurements, indicating magneto-elastic coupling, i.e. an effect on the crystal structure of the magnetic ordering. Lattice parameter *a* contracts faster below *T*_N_, and *c* remains approximately flat below *T*_N_ instead of undergoing continued thermal contraction. These observations are consistent with in-plane ferromagnetic coupling and between-plane antiferromagnetic coupling. A variable temperature PND measurement on the same sample of KCoNiSe_2_ revealed that the magnetic Bragg peaks appear below the Néel temperature of 175 K established from the magnetometry measurements (Figure 10b).

**Figure 10:**
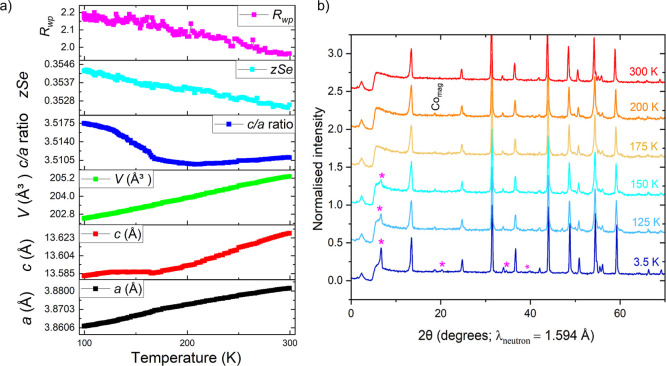
(a) Variable-temperature (100–300 K) PXRD of KCoNiSe_2_. (b) Variable-temperature PND measurement of KCoNiSe_2_ showing observed magnetic Bragg peaks with magenta asterisks.

Our magnetometry results confirm ferromagnetic order below 90 K in KCo_2_Se_2_ (Figure 11b,c).^2^ There were no additional reflections observed for KCo_2_Se_2_ in the PND pattern measured at 3.5 K (Figure 11a). Inclusion of ferromagnetically arranged moments on the Co ions, in a magnetic phase similar to that reported for RbCo_2_Se_2_ from single crystal neutron diffraction,^7^ suggested a similar small ferromagnetic moment of 0.6(1) μ_B_ per Co, although this small moment is at the edge of what could be obtained from our data. As Co is replaced by Ni, there is evidently a changeover in the coupling between layers from ferromagnetic to antiferromagnetic, and this is presumably mainly a consequence of the increasing electron count.^14^

**Figure 11:**
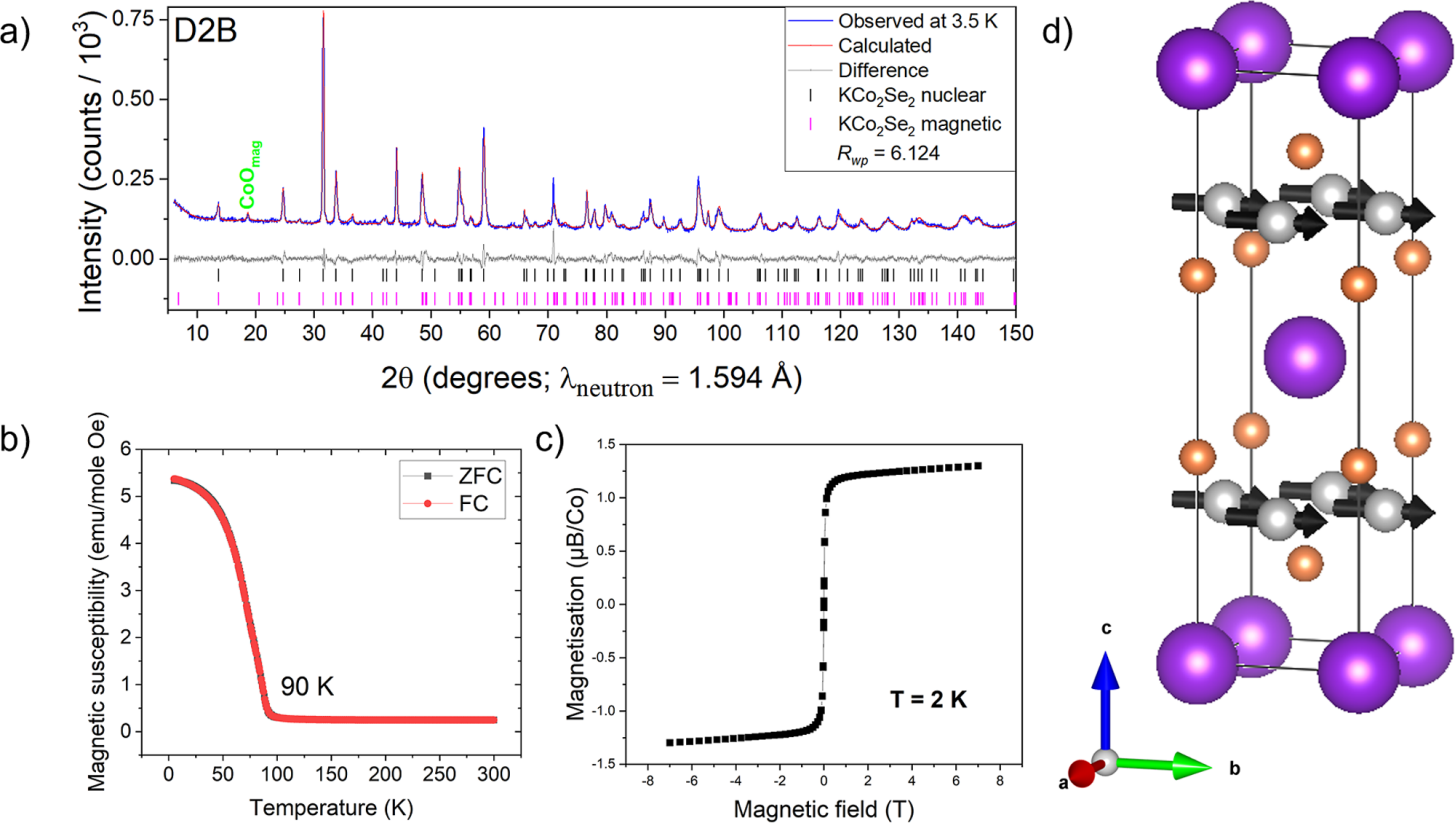
(a) Rietveld refinement of PND of KCo_2_Se_2_ at 3.5 K. (b) ZFC-FC measurement of KCo_2_Se_2_ showing a ferromagnetic transition at 90 K. (c) M v H isotherm measured at 2 K for KCo_2_Se_2_. (d) Magnetic structure of KCo_2_Se_2_. KCo_2–*x*_Ni_*x*_S_2_ series.

The members of the sulfide solid solution were more difficult to synthesize with high purity than the selenide analogues. In particular Co_9_S_8_ and K_2_Ni_3_S_4_ were found as competing transition-metal-containing impurities. The changes in lattice parameters with *x* in KCo_2–*x*_Ni_*x*_S_2_ (Figure S13) follow similar trends to those of the selenide analogues. Likewise, magnetometry of KCo_2–*x*_Ni_*x*_S_2_ reveals similar magnetic behavior to the selenide analogues with the end members KCo_2_S_2_ and KNi_2_S_2_ behaving as ferromagnetic and paramagnetic metals,^9,34^ respectively, and antiferromagnetism observed for 0.5 ≤ *x* ≤ 1.5. Magnetic susceptibilities for the KCo_2–*x*_Ni_*x*_S_2_ solid solution are shown in Figure 12a and the magnetic phase diagram in Figure 12b is comparable to that of the selenides.

**Figure 12:**
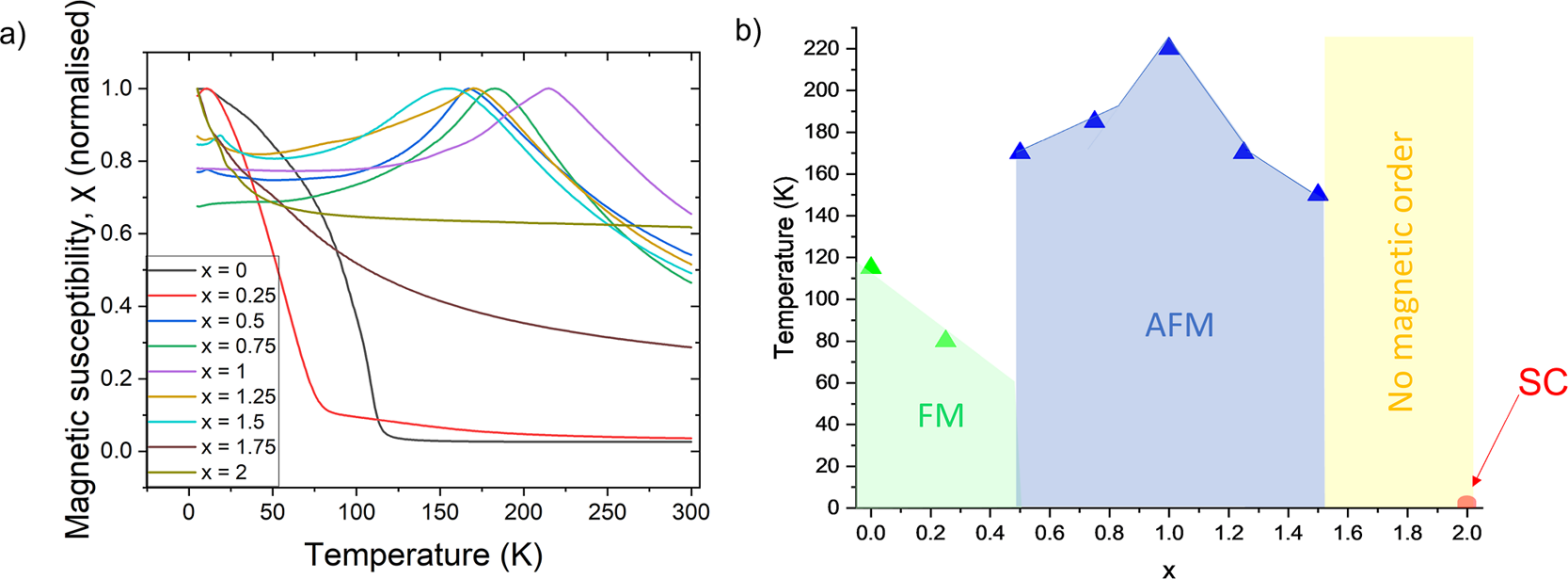
(a) Magnetic susceptibility (ZFC measurements) as a function of temperature for the series KCo_2–*x*_Ni_*x*_S_2_, 0 ≤ *x* ≤ 2. (b) Magnetic phase diagram for KCo_2–*x*_Ni_*x*_S_2_ (0 ≤ *x* ≤ 2) showing the ferromagnetic region (green), antiferromagnetic region (blue), paramagnetic region (yellow), and superconducting region (red).

### Magnetic Ordering in KCo_2–*x*_Ni_*x*_S_2_

Three antiferromagnetic members of the series, KCo_0.5_Ni_1.5_S_2_, KCoNiS_2_, and KCo_1.5_Ni_0.5_S_2_, were measured using PND on the WISH beamline at ISIS. Low temperature (1.5 K) PND measurements reveal (003), (005), (203), (025) and (207) Bragg reflections (which have zero intensity in the body-centered tetragonal nuclear structure) in KCoNiS_2_ (Figure 13a) and KCo_1.5_Ni_0.5_S_2_ (Figure 13b). In KCo_0.5_Ni_1.5_S_2_ these reflections were not evident (Figure S14) suggesting that the rather broader susceptibility maximum in this compound (Figure 12a) is not associated with the long-range magnetic ordering which can be probed by diffraction. Like in the selenide analogues, the magnetic structure in the long-range ordered cases can be described as A-type antiferromagnetic in orthorhombic space group *Cmcm* (63.468 in the BNS scheme) as described above for the selenides with long-range ordered moments of Co/Ni ions of 1.22(2) μ_B_ in KCoNiS_2_ and 1.06(2) μ_B_ in KCo_1.5_Ni_0.5_S_2_. PND patterns measured around room temperature are shown in Figures S15–S17.

**Figure 13:**
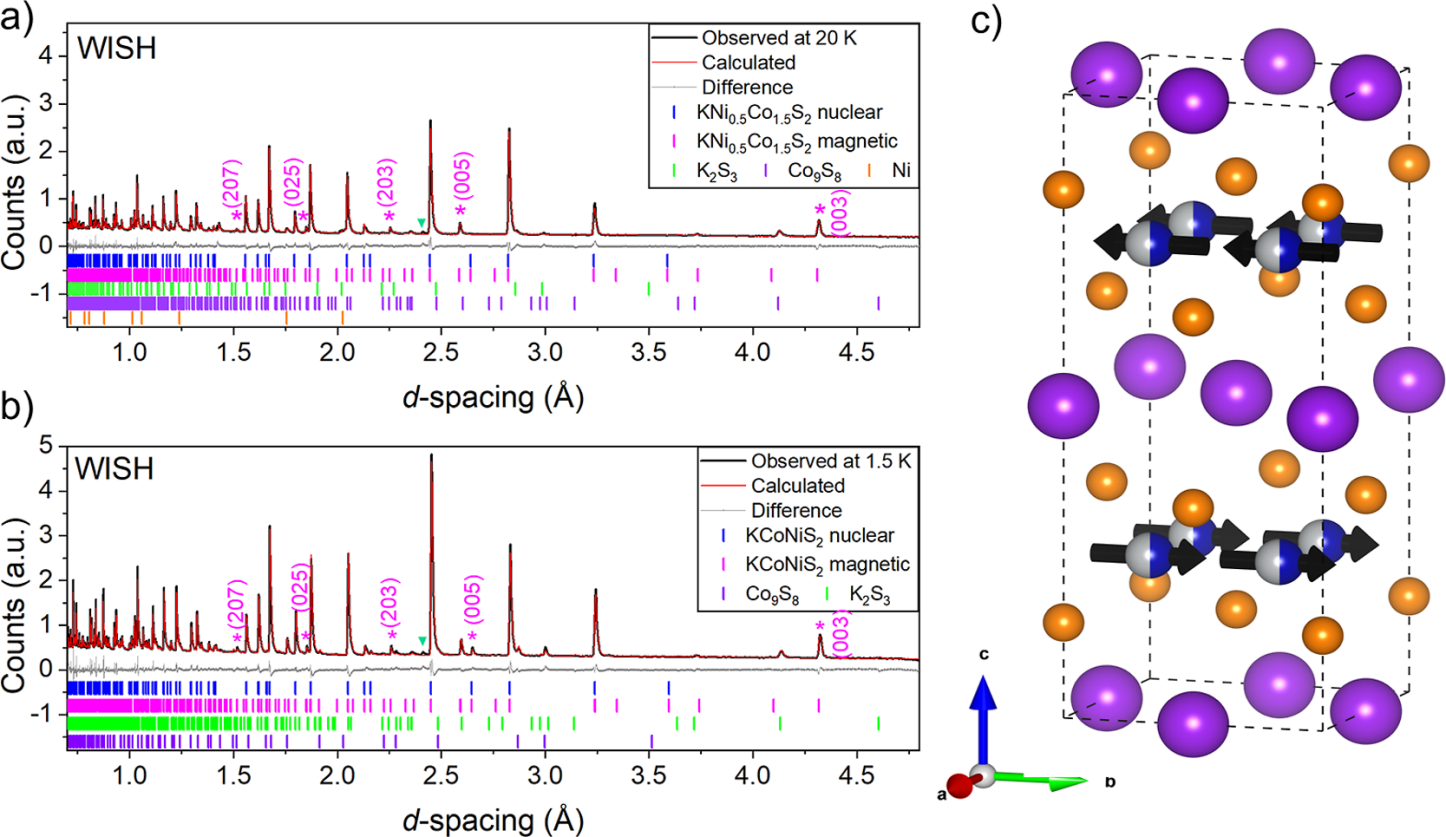
(a) Rietveld refinement of KCo_1.5_Ni_0.5_S_2_ against PND data measured at 20 K, *R*_wp_ = 5.28% and χ^2^ = 1.02 (b) Rietveld refinement of KCoNiS_2_ against PND data measured at 1.5 K, *R*_wp_ = 6.28% and χ^2^ = 0.97. (c) Magnetic structure for KCoNiS_2_ and KCo_1.5_Ni_0.5_S_2_, with ferromagnetic coupling of Co/Ni moments within the *ab* plane and antiferromagnetic coupling along the stacking axis (*c* direction). Purple asterisks mark magnetic reflections indexed on the *Cmcm* cell which are not present at room temperature. The green triangle represents an unknown impurity.

A variable temperature PXRD measurement was carried out on KCo_1.5_Ni_0.5_S_2_ between 100 and 300 K to monitor the changes in lattice parameters and unit cell volume (Figure 14a). As in the selenide analogues, nonlinear trends were observed for the *a* and *c* lattice parameters and for the *c*/*a* ratio which increases sharply below *T*_N_ (185 K). A variable-temperature PND measurement on the same sample of KCo_1.5_Ni_0.5_S_2_ revealed that the magnetic Bragg peaks appear just below the Néel temperature of 185 K deduced from the magnetometry. The (003), (005), and (203) magnetic reflections are visible from below 180 K (Figure 14b).

**Figure 14:**
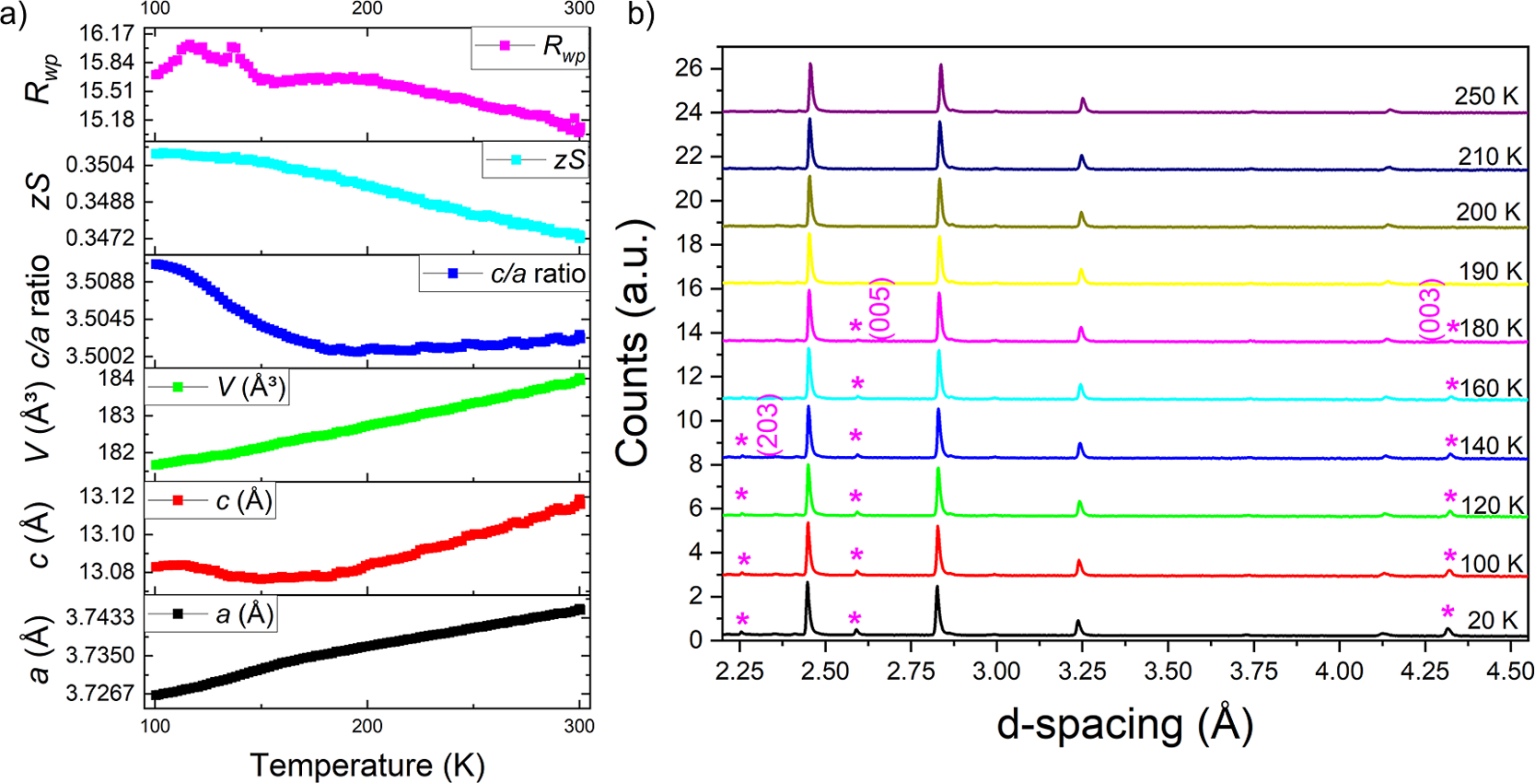
(a) Variable-temperature (100–300 K) PXRD of KCoNiS_2_. (b) Variable-temperature PND measurement of KCo_1.5_Ni_0.5_S_2_ showing magnetic Bragg peaks (purple asterisks).

### Structure and Magnetism of CsCo_2–*x*_Ni_*x*_Se_2_

A analogous solid solution to those described above was explored with the larger Cs^+^ cation replacing the K^+^ cation. The changes in lattice parameters with *x* are summarized in Figure S18, and a Rietveld refinement of CsCoNiSe_2_ (*x* = 1), against high-resolution X-ray data is shown in Figure S19. Magnetometry indicated antiferromagnetism for 0 ≤ *x* ≤ 1.75 (Figure 15a,b) with no evidence for ferromagnetism in the Co-rich members of the series, although high-field measurements^2^ show that CsCo_2_Se_2_ has a metamagnetic transition at around 3 T at 2 K which may indicate a transition to the fully ferromagnetic state. Previously the CsNi_2_Se_2_ end member has been shown to display paramagnetic behavior down to low temperatures.^35^ The magnetic structure of CsCoNiSe_2_ was then probed with PND at 3.5 K (Figure 16a) and 300 K (Figure 16b). Like in the analogous compounds discussed above, the magnetic Bragg peaks were accounted for by the A-type antiferromagnetic model in space group *Cmcm*, with a refined long-range ordered moment of 0.80 μ_B_ per transition metal ion.

**Figure 15:**
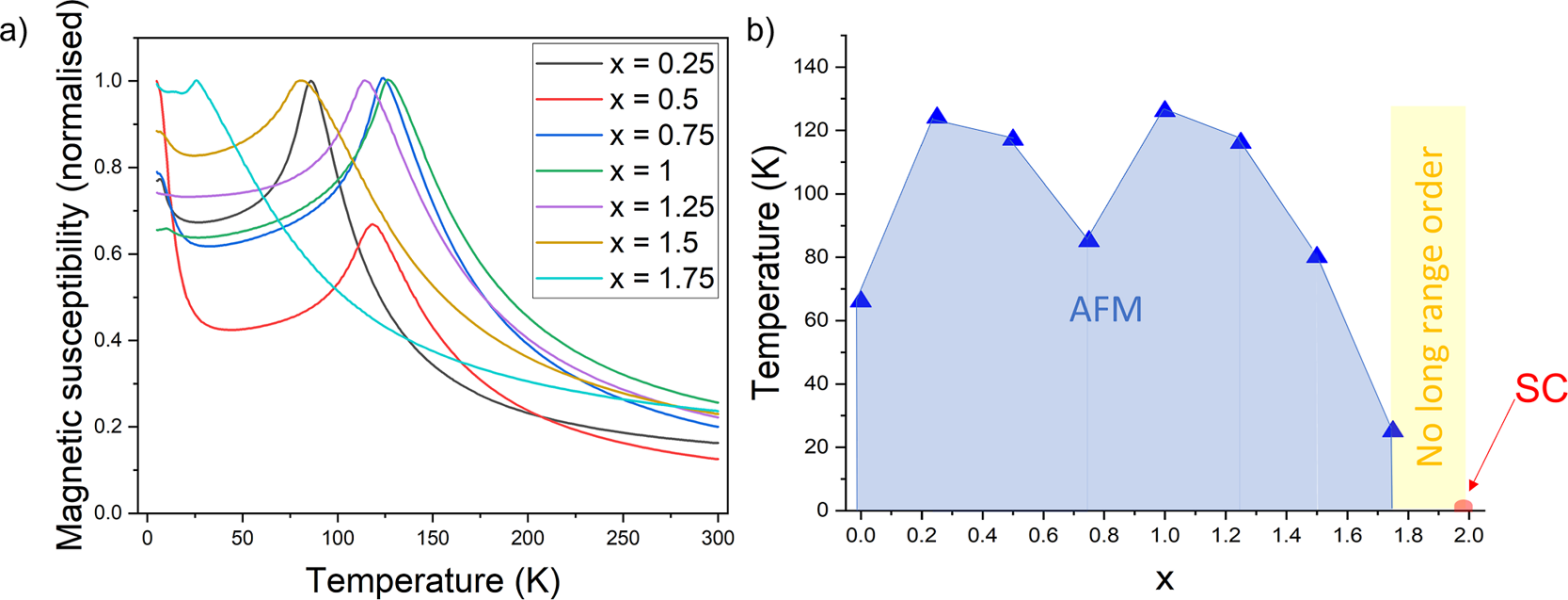
(a) Magnetic susceptibility (ZFC measurements) as a function of temperature for the series CsCo_2–*x*_Ni_*x*_Se_2_, 0.25 ≤ *x* ≤ 1.75. (b) Magnetic phase diagram for CsCo_2–*x*_Ni_*x*_Se_2_, 0.25 ≤ *x* ≤ 1.75.

**Figure 16:**
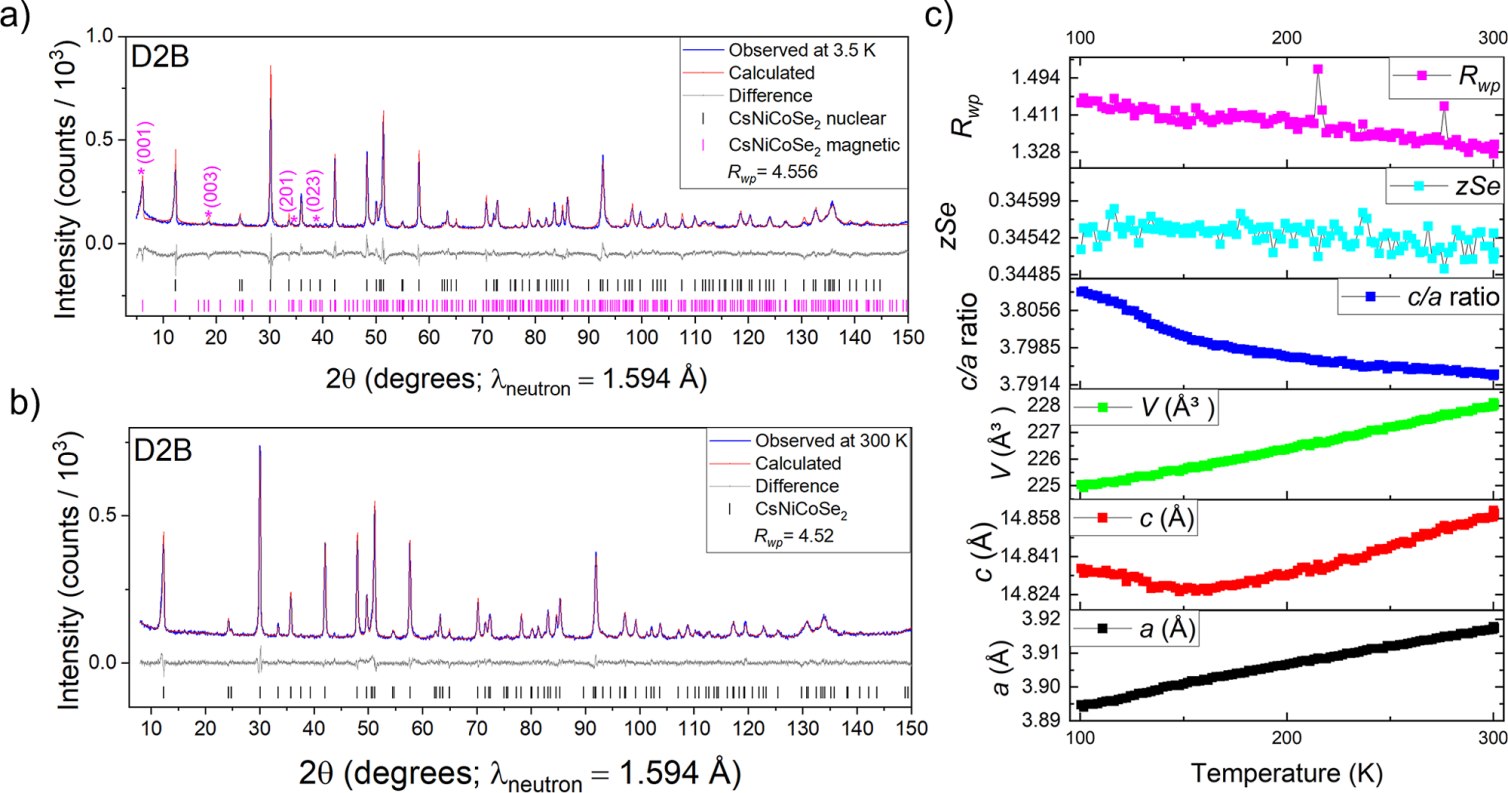
(a) Rietveld refinement of CsCoNiSe_2_ against PND data measured at 3.5 K, *R*_wp_ = 5.53% and χ^2^ = 1.62. (b) Rietveld refinement of CsCoNiSe_2_ against PND data measured at 300 K, *R*_wp_ = 4.52% and χ^2^ = 1.45. (c) Results from variable-temperature (100–300 K) PXRD of CsCoNiSe_2_.

Variable temperature PXRD measurement on CsCoNiSe_2_ between 100 and 300 K showed similar nonlinear trends in *a* and *c* lattice parameters as for the analogous members of the KCo_2–*x*_Ni_*x*_S_2_ and KCo_2–*x*_Ni_*x*_Se_2_ solid solutions, consistent with the A-type antiferromagnetism (Figure 16c).

## Conclusions

Three solid solutions of the ThCr_2_Si_2_-type structure were synthesized, and their magnetic properties were analyzed. KCo_2–*x*_Ni_*x*_Se_2_, KCo_2–*x*_Ni_*x*_S_2_, and CsCo_2–*x*_Ni_*x*_Se_2_ solid solutions were synthesized with 0 ≤ *x* ≤ 2, and synchrotron PXRD reveals linear trends in lattice parameters. Magnetometry measurements on the two K-based systems show drastic changes in magnetism: addition of Ni causes change from long-range ferromagnetic to antiferromagnetic ordering at *x* = 0.5, while further Ni-substitution gives rise eventually to Ni-rich compositions that do not exhibit magnetic long-range order. Neutron powder diffraction revealed A-type antiferromagnetism in KCoNiSe_2_, KCo_1.5_Ni_0.5_Se_2_ (and their sulfide analogues) and CsCoNiSe_2_: ferromagnetic coupling within (Co/Ni)Se and antiferromagnetic coupling between layers. The PND results show that there is no long-range chemical order of the Ni and Co species within the chalcogenide layers, and the chemical disorder within these layers does not suppress long-range magnetic order. Our results suggest that the reasons for drastic changes in magnetism across the series are due to the addition of an extra electron per Ni to the 3d band of the system.

## Supplementary Material



## Notes

Further tables of structural refinement parameters and figures showing further Rietveld refinements (.pdf) and magnetometry and SEM–EDX results accompany this paper (PDF)
