# Correction to “Competing Magnetism in Layered Mixed Transition Metal Chalcogenides KCo_2–*x*_Ni_*x*_Se_2_, KCo_2–*x*_Ni_*x*_S_2_, and CsCo_2–*x*_Ni_*x*_Se_2_”

**DOI:** 10.1021/acs.chemmater.5c02062

**Published:** 2025-08-14

**Authors:** Ludmila Taskesen, Robert D. Smyth, Lemuel E. Crentsil, James I. Murrell, Emmanuelle Suard, Pascal Manuel, Simon J. Clarke

In preparation of the manuscript, the two samples with *x* = 0.25 and *x* = 0.75 in Figure 15(a) were inadvertently transposed in Figure 15(b). The corrected Figure 15 is shown below, where panel (a) is unchanged and panel (b) has been amended.

**Figure 15:**
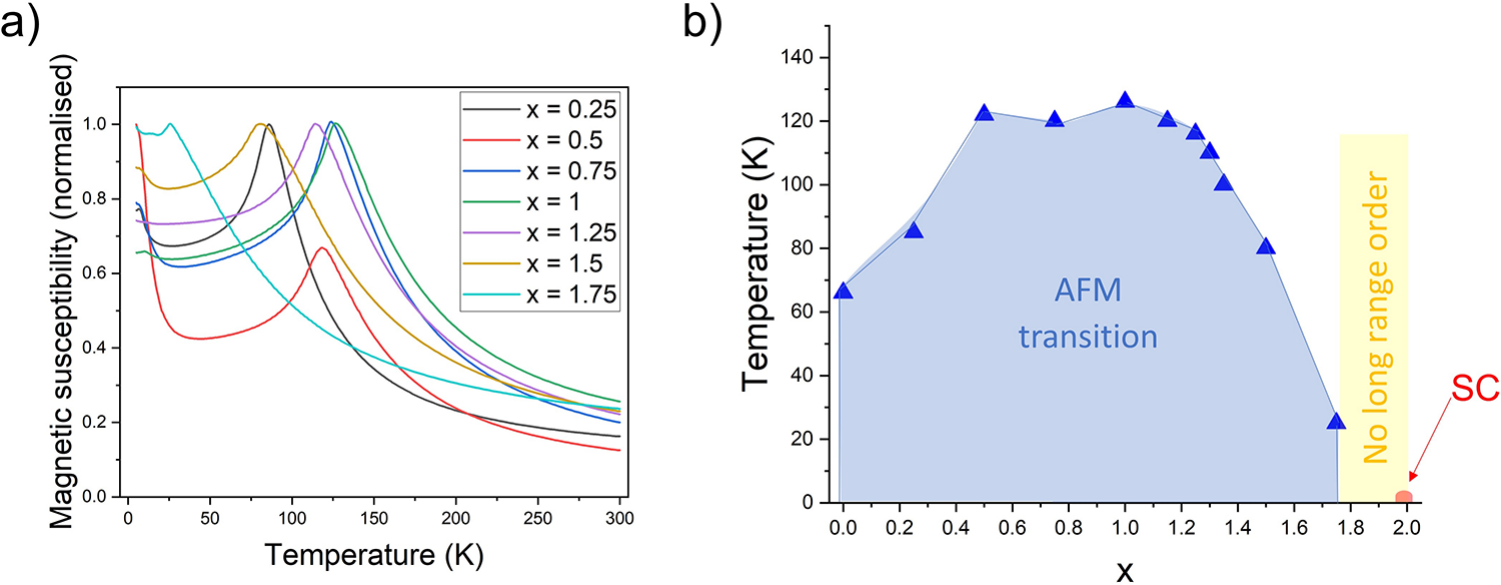
(a) Magnetic susceptibility (ZFC measurements) as a function of temperature for the series CsCo_2–*x*_Ni_*x*_Se_2_, 0.25 ≤ *x* ≤ 1.75. (b) Magnetic phase diagram for CsCo_2–*x*_Ni_*x*_Se_2_, 0.25 ≤ *x* ≤ 1.75.

