# Supplementary material for: Competing Magnetism in Layered Mixed Transition Metal Chalcogenides KCo2–x Ni x Se2, KCo2–x Ni x S2, and CsCo2–x Ni x Se2
Source: Chem Mater. 2025 Jul 11;37(14):5300–11. doi: 10.1021/acs.chemmater.5c00996 (PMC12288000; doi:10.1021/acs.chemmater.5c00996)
Supplement: Supplementary file 1 [file cm5c00996_si_001.pdf]

Competing magnetism in layered mixed transition metal chalcogenides $KCo_{2-x}Ni_xSe_2$, $KCo_{2-x}Ni_xS_2$, and $CsCo_{2-x}Ni_xSe_2$

Ludmila Taskesen[1], Robert D. Smyth[1], Lemuel E. Crentsil[1], James I. Murrell[1], Emmanuelle Suard[3], Pascal Manuel[4], Simon J. Clarke[1]*

[1] Department of Chemistry, University of Oxford, Inorganic Chemistry Laboratory, South Parks Road, Oxford OX1 3QR, UK.

[3] Institut Laue-Langevin (ILL), BP 156, 71 Avenue des Martyrs, Grenoble 38042, France.

[4] ISIS Facility, Rutherford Appleton Laboratory, Harwell Oxford, Didcot, OX1 10QX, U.K.

*email address: simon.clarke@chem.ox.ac.uk

Supporting Information

Table S1. Structural parameters for $KNiCoSe_2$ from Rietveld refinement against I11 RT data (Diamond Light Source) and D2B RT data (ILL).

Diffractometer	I11	D2B
Radiation	X-ray	Neutron
Wavelength (Å)	0.824619	1.594
Temperature (K)	298	300
R_{wp} (%)	1.778	3.03914
χ^2	4.39	1.46(3)
Space group	*I*4/*mmm*	*I*4/*mmm*
a (Å)	3.88427(1)	3.88292(4)
c (Å)	13.6133(1)	13.6177(3)
V (Å^3)	205.391(1)	205.318(7)
z(Se)	0.35404(6)	0.35411(6)
ρ (g cm^{-3})	5.08779	5.05672
Ni/Co–Se (Å)	2.4038(5)	2.4036(9)
Se–Se (Å)	3.974(1)	3.975(3)
Ni/Co–Ni/Co (Å)	2.74657(1)	2.74565(1)
Se–Ni/Co–Se (°)	107.87(2)	107.80(2)
U_{11} (K) (Å^2)	0.00473(9)	0.026(2)
U_{33} (K) (Å^2)	0.020(1)	0.016(3)
U_{11} (Ni) (Å^2)	0.0002(4)	0.0163(1)
U_{33} (Ni) (Å^2)	0.0038(7)	0.015(1)
U_{11} (Co) (Å^2)	0.0002(4)	0.0163(1)
U_{33} (Co) (Å^2)	0.0038(7)	0.015(1)
U_{11} (Se) (Å^2)	0.00014(3)	0.0098(6)
U_{33} (Se) (Å^2)	0.00165(5)	0.0093(1)

Table S2. Unit cell parameters, interatomic distances and smaller tetrahedral angle (four-fold) in $KCo_{2-x}Ni_xSe_2$

	***x* = 0**	***x* = 0.25**	***x*= 0.5**	***x* = 0.75**	***x* = 1**	***x* = 1.25**	***x* = 1.5**	***x* = 1.75**	***x* = 2**
***a* (Å)**	3.8443(1)	3.8413(1)	3.8539(1)	3.8578(1)	3.8843(1)	3.8903(1)	3.8969(1)	3.8925(1)	3.9066(1)
***c* (Å)**	13.7722(4)	13.8176(1)	13.7555(1)	13.7436(2)	13.6133(1)	13.5707(2)	13.5326(1)	13.4457(2)	13.4324(4)
***V* (Å³)**	203.534(5)	203.881(6)	204.304(4)	204.549(3)	205.391(1)	205.389(2)	205.510(5)	205.511(3)	205.000(9)
**$d_{X\text{-}X}$ (Å)**	4.021(3)	4.034(5)	4.016(3)	4.013(1)	3.974(1)	3.962(2)	3.951(9)	3.9261(7)	3.9223(2)
**$d_{T\text{-}X}$ (Å)**	2.3971(4)	2.3987(4)	2.3999(1)	2.4008(3)	2.4038(5)	2.4038(5)	2.4036(6)	2.3965(9)	2.4014(4)
**$d_{T\text{-}T}$ (Å)**	2.7183(1)	2.7181(3)	2.7251(2)	2.7279(8)	2.7466(1)	2.7509(2)	2.7556(3)	2.7524(4)	2.7624(3)
**$\delta_{X\text{-}T\text{-}X}$ (°)**	106.62(4)	106.39(2)	106.82(5)	106.92(5)	107.87(2)	108.07(7)	108.32(1)	108.61(3)	108.86(1)

Table S3. Experimental details of Single crystal X-ray diffraction experiment on KNi_2Se_2

The structural data extracted from Single crystal XRD experiment agrees at the 3σ level with literature values for KNi_2Se_2 reported by Lei et al. (ref. 12 in the main article). The refined chemical formula in Lei et al.'s single crystal study conducted at room temperature was reported as $K_{0.95(1)}Ni_{1.86(2)}Se_{2.00(1)}$. Lattice parameter *a* was reported to be 3.8707(5) Å and *c* as 13.591(4) Å, while the unit cell volume was 203.62(7) $Å^3$.

Crystal data	
Chemical formula	$K_{0.90(3)}Ni_{1.948(14)}Se_{2.00(9)}$
M_r	310.3
Crystal system, space group	Tetragonal, *I*4/*mmm*
Temperature (K)	150
a, *c* (Å)	3.8709 (13), 13.583 (5)
V ($Å^3$)	203.53 (12)
Z	2
Radiation type	Mo *K*α
Crystal size (mm)	0.05 × 0.04 × 0.01
Data collection	
Diffractometer	(Rigaku) Oxford Diffraction SuperNova A
T_{min}, T_{max}	0.31, 1
No. of measured, independent and observed [$I > 3\sigma(I)$] reflections	1504, 121, 103
R_{int}	0.123
Refinement	
$R[F^2 > 2\sigma(F^2)]$, $wR(F^2)$, S	0.058, 0.146, 4.44
No. of reflections	121
No. of parameters	9
$\Delta\rho_{max}$, $\Delta\rho_{min}$ (e $Å^{-3}$)	1.59, −1.23

Table S3 cont. Structural details for Single crystal X-ray diffraction experiment on KNi_2Se_2

Atom	Wyckoff site	*x*	*y*	*z*	Occupancy	U_{eq}/U_{iso}(Å^2)
K1	2*a*	0	0	0	0.90(3)	0.022(2)
Ni1	4*d*	0	0.5	0.25	0.947(7)	0.0185(8)
Se1	4*e*	0	0	0.35402(18)	1	0.0166(7)

Anisotropic displacement factor harmonic parameters

Atom	U_{11} (Å^2)	U_{22}(Å^2)	U_{33}(Å^2)	U_{12}(Å^2)	U_{13}(Å^2)	U_{23}(Å^2)
K1	0.024 (3)	0.024 (3)	0.019 (5)	0	0	0
Ni1	0.0135(11)	0.0135 (11)	0.0285 (19)	0	0	0
Se1	0.0114 (9)	0.0114 (9)	0.0270 (15)	0	0	0

Note: off-diagonal terms are zero by symmetry

Table S4. Atomic occupancies of $KCo_{2-x}Ni_xSe_2$ series before scaling up from K_2Se standard, where both K and TM (Co/Ni) were scaled up.

**Nominal Composition**	**Observed composition**	**K**	**Co**	**Ni**	**Se**
KCo_2Se_2	$K_{0.78(6)}Co_{1.90(7)}Se_{2.0(1)}$	0.78 ± 0.06	1.9 ± 0.07	-	2.0 ± 0.01
$KCo_{1.75}Ni_{0.25}Se_2$	$K_{0.76(2)}Co_{1.55(7)}Ni_{0.17(2)}Se_{2.0(1)}$	0.76 ± 0.02	1.55 ± 0.07	0.17 ± 0.02	2.00 ± 0.01
$KCo_{1.5}Ni_{0.5}Se_2$	$K_{0.79(3)}Co_{1.31(8)}Ni_{0.42(3)}Se_{2.0(1)}$	0.79 ± 0.03	1.31 ± 0.08	0.42 ± 0.03	2.00 ± 0.01
$KCo_{1.25}Ni_{0.75}Se_2$	$K_{0.79(2)}Co_{1.1(1)}$ $Ni_{0.70(5)}Se_{2.0(1)}$	0.79 ± 0.02	1.10 ± 0.01	0.7 ± 0.05	2.00 ± 0.01
$KCoNiSe_2$	$K_{0.79}Co_{1.05(6)}Ni_{0.91(6)}Se_{2.0}$	0.79 ± 0.05	1.05 ± 0.06	0.91 ± 0.06	2.00 ± 0.01
$KCo_{0.75}Ni_{1.25}Se_2$	$K_{0.87(2)}Co_{0.74(6)}Ni_{1.09(7)}Se_{2.0(1)}$	0.87 ± 0.02	0.74 ± 0.06	1.09 ± 0.07	2.00 ± 0.01
$KCo_{0.5}Ni_{1.5}Se_2$	$K_{0.90(4)}Co_{0.40(4)}$ $Ni_{1.3(1)}Se_{2.0(2)}$	0.90 ± 0.04	0.40 ± 0.04	1.30 ± 0.01	2.00 ± 0.02
KNi_2Se_2	$K_{0.85(6)}Ni_{1.79(9)}Se_{2.00(9)}$	0.85 ± 0.06	-	1.79± 0.09	2.00 ± 0.09

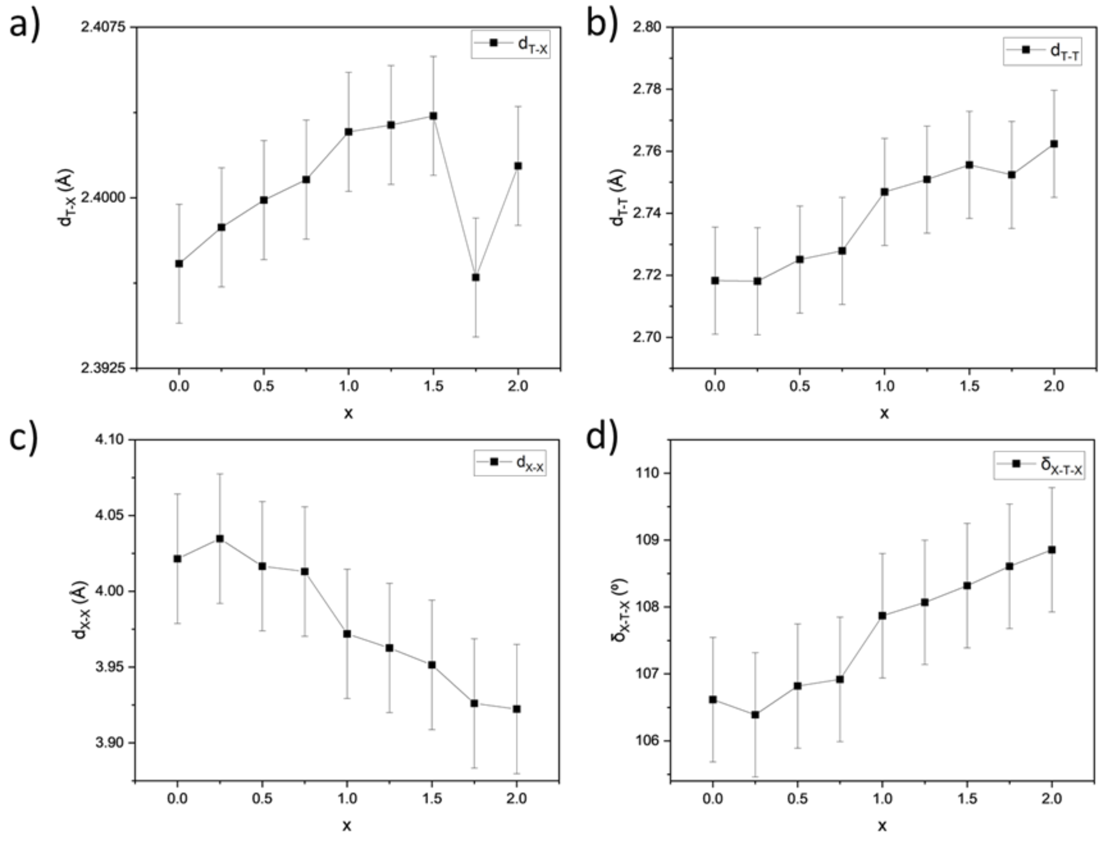

Figure S1. Changes in interatomic distances and angles across the series; a) Co/Ni–Se distance, b) Co/Ni–Co/Ni distance, c) Se–Se distance, d) tetrahedral angle Se–Co/Ni–Se, extracted from Rietveld refinement of I11 high resolution data (Diamond Light Source). The *X–T–X* angle is the angle defined in Figure 2(c) with a multiplicity of 4.

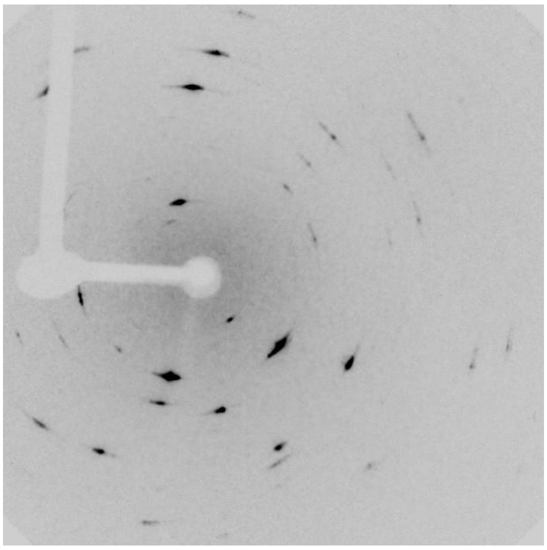

Figure S2. Sample single crystal diffraction frame (CCD image) of $KCoNiSe_2$ at 150 K showing elongated spots.

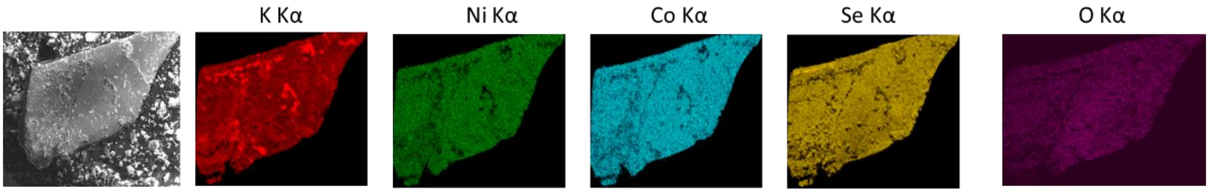

Figure S3. SEM image of $KCoNiSe_2$ single crystal. There are some potassium-rich regions shown in bright red, which do not contain any other elements, including oxygen. It is clear from the map data and from specific points that there is some surface oxidation occurring on transfer to the instrument which led to samples being air-exposed for < 2 minutes.

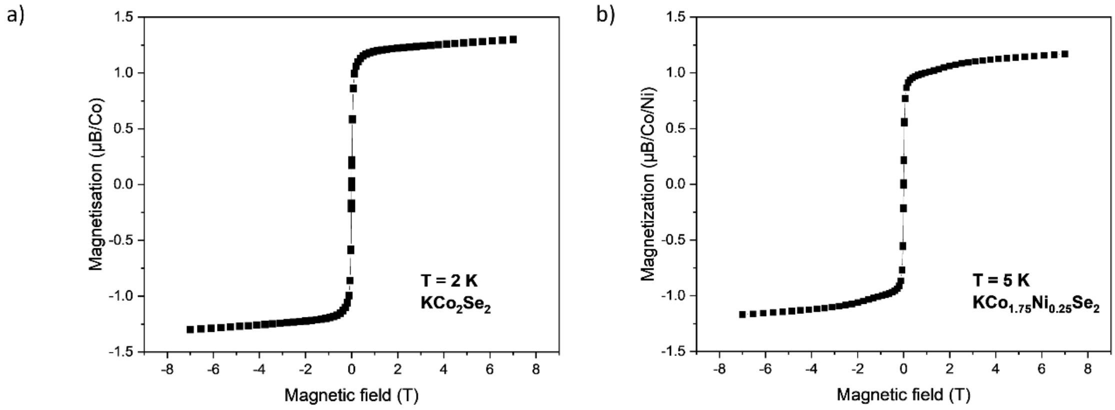

Figure S4. Magnetisation isotherms for a) KCo_2Se_2 (x = 0), and b) $KCo_{1.75}Ni_{0.25}Se_2$ (x = 0.25) measured at 2K and 5K, respectively.

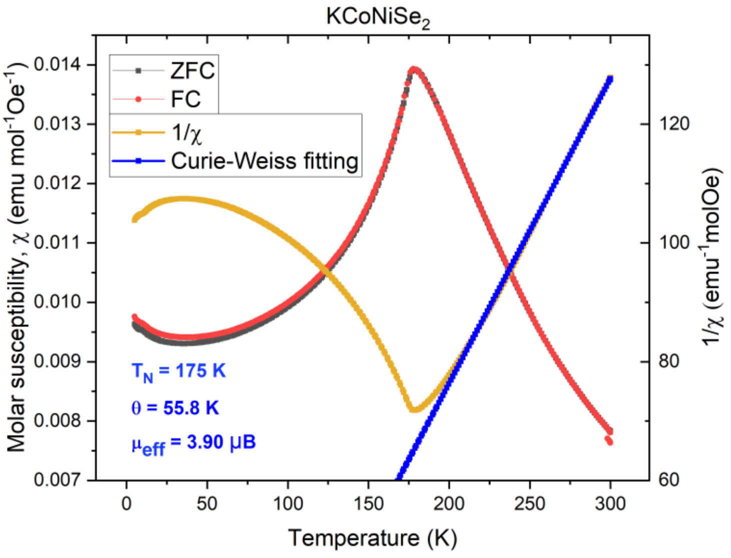

Figure S5. Attempted Curie-Weiss fitting of $KCoNiSe_2$. For the antiferromagnetic samples the linear portion of the plot of the variation of $1/\chi$ with temperature above the Néel temperature was fitted to the Curie-Weiss law, $\chi = C/(T-\theta)$ for $KCoNiSe_2$. Using this treatment, the effective moment per transition metal ion, $\mu_{eff}^2 = 8C_m$, was calculated for the antiferromagnetic members of the series, listed in Table S5, together with values of T_N and θ (although see note below about the validity of this approach).

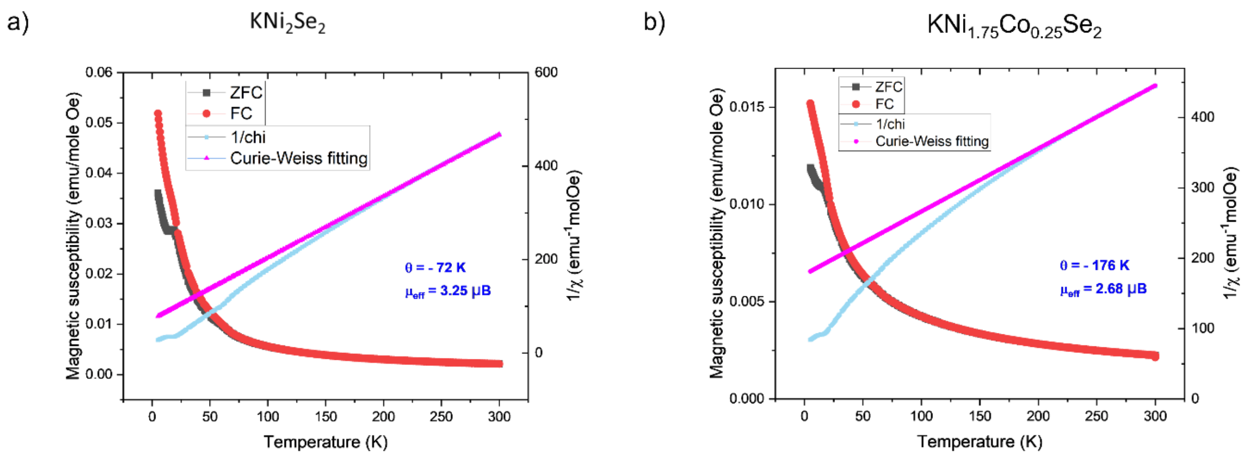

Figure S6. Modified Curie-Weiss fits of paramagnetic members of the series a) KNi_2Se_2 (x = 0) and b) $KNi_{1.75}Co_{0.25}Se_2$ (x = 0.25). The modification is to include a temperature-independent paramagnetic contribution, as the high temperature part of $1/\chi$ vs T plot was not linear and deviated from the Curie-Weiss law.

Table S5. Summary of physical properties of $KCo_{2-x}Ni_xSe_2$ obtained from magnetometry (T_N, θ, and μ_{eff}) and neutron powder diffraction. The Weiss temperature, θ, which gives an estimate of the average strength of the coupling between magnetic moments, is positive for antiferromagnetic members of the series, which is consistent with the strong in-plane ferromagnetism and the weaker coupling between the layers which eventually drives long-range A-type magnetic ordering. For the members of the series with higher Ni contents which do not show evidence for magnetic long-range order, the Weiss temperatures are negative, suggesting that antiferromagnetic interactions dominate. We should note that although these compounds appear to obey the Curie-Weiss or modified (with a constant contribution to the susceptibility) Curie-Weiss law, the Weiss temperatures are not much smaller than the temperatures in the range chosen for the fitting, and the effective moments are very much larger than the long-range ordered moments obtained from powder neutron diffraction. The results obtained from this treatment should be interpreted with some caution.

**Composition**	***x***	**T_N (K)**	**θ(K)**	**μ_{eff} (μB)**	**Long-range ordered moment per T ion (μ_B)**
KCo_2Se_2	0	90 (FM)	-	-	-
$KCo_{1.75}Ni_{0.25}Se_2$	0.25	65 (FM)	-	-	-
$KCo_{1.5}Ni_{0.5}Se_2$	0.5	150	69	4.34	1.07
$KCo_{1.25}Ni_{0.75}Se_2$	0.75	145	49	4.85	-
$KCoNiSe_2$	1	175	55.8	3.90	1.18
$KCo_{0.75}Ni_{1.25}Se_2$	1 .25	166	29.5	3.18	-
$KCo_{0.5}Ni_{1.5}Se_2$	1.5	120	-37.7	3.32	-
$KCo_{0.25}Ni_{1.75}Se_2$	1.75	-	-176	2.68	-
KNi_2Se_2	2	-	-72	3.25	-

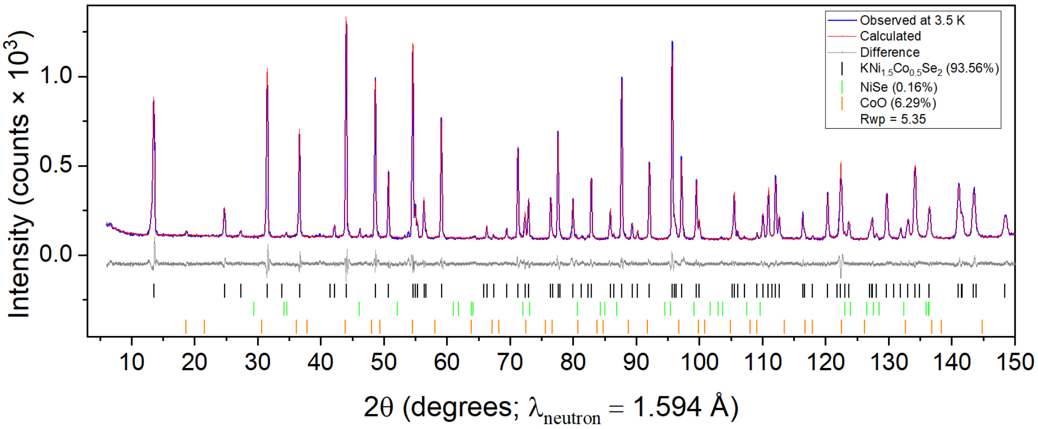

Figure S7. Rietveld refinement of $KCo_{0.5}Ni_{1.5}Se_2$ against PND data measured at 3.5 K at D2B, R_{wp} = 5.35% and χ^2 = 2.00.

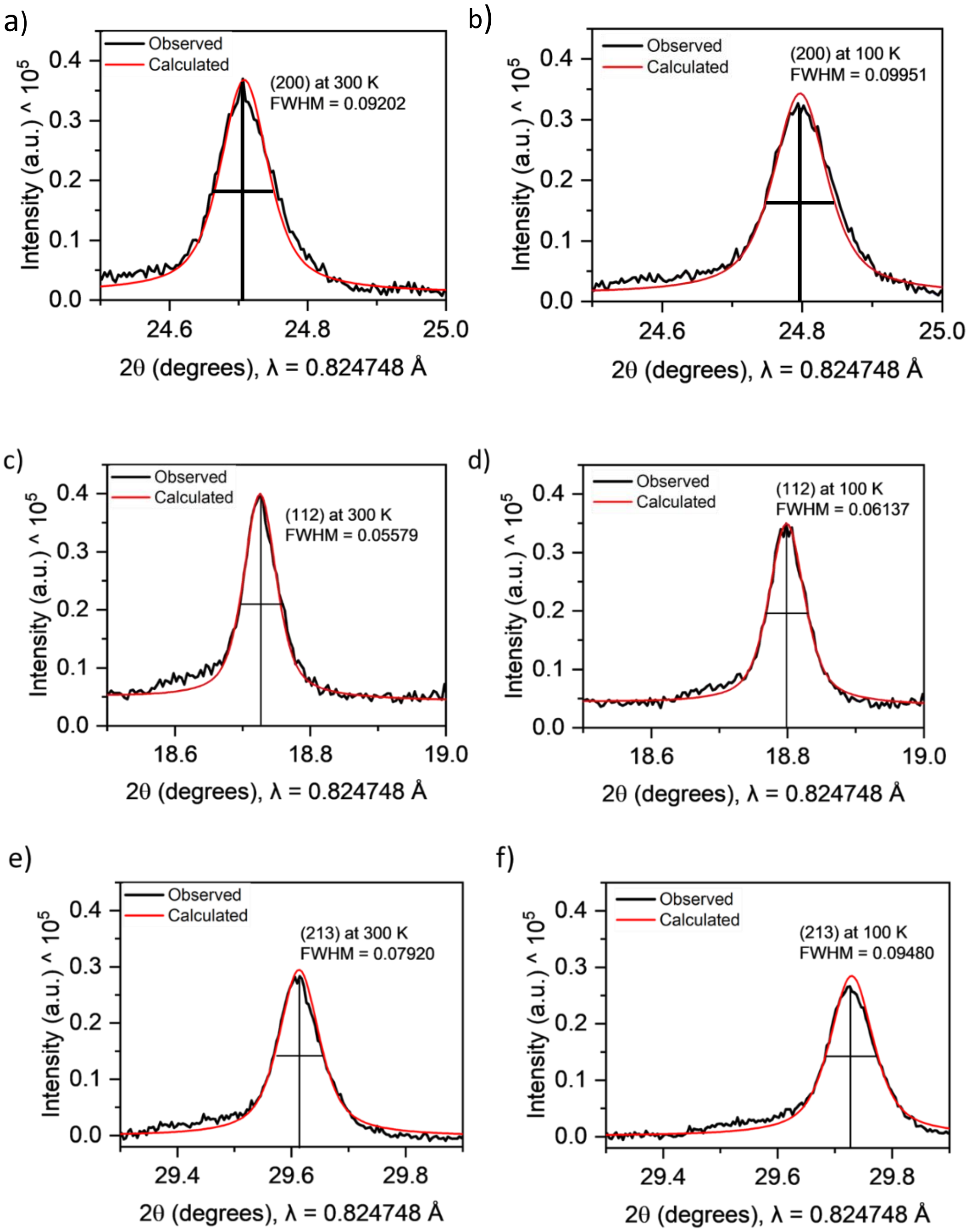

Figure S8. Full width at half maximum (FWHM) calculation of the Gaussian fit of a) 200 peak at 300 K and b) 100 K, c) 112 peak at 300 K and d) 100 K, e) 213 peak at 300 K and f) 100 K. The high-resolution data were collected on instrument I11 at Diamond Light Source.

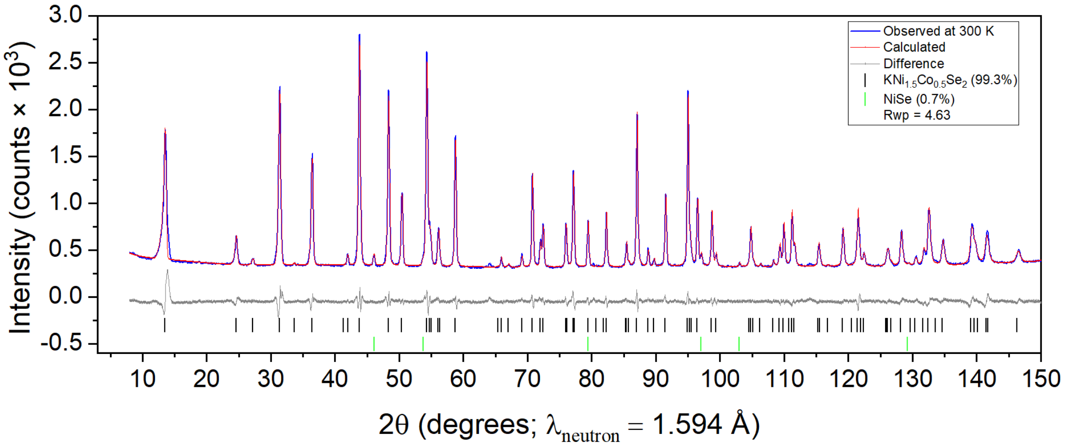

Figure S9. Rietveld refinement of $KCo_{0.5}Ni_{1.5}Se_2$ against PND data measured at 300 K on D2B, R_{wp} = 4.63% and χ^2 = 1.79.

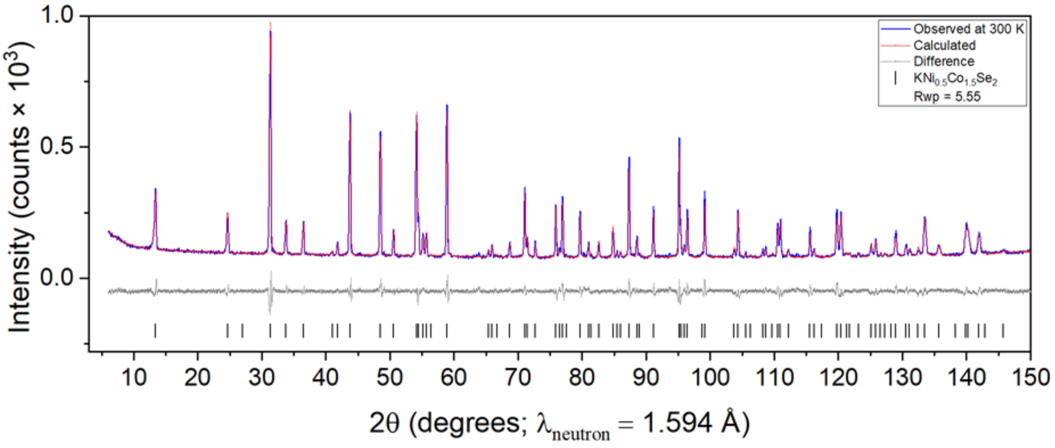

Figure S10. Rietveld refinement of $KCo_{1.5}Ni_{0.5}Se_2$ against PND data measured at 300 K on D2B, R_{wp} = 5.55% and χ^2 = 1.81.

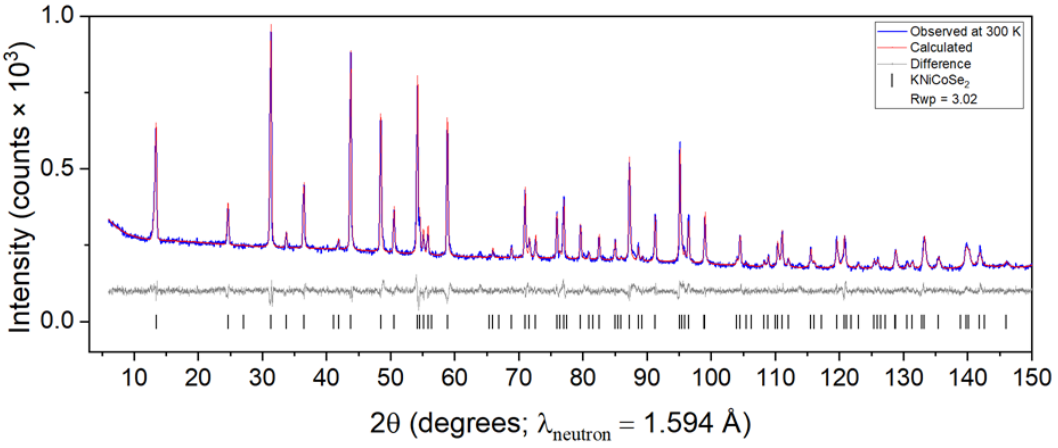

Figure S11. Rietveld refinement of $KCoNiSe_2$ against PND data measured at 300 K on D2B, R_{wp} = 3.02% and χ^2 = 1.45.

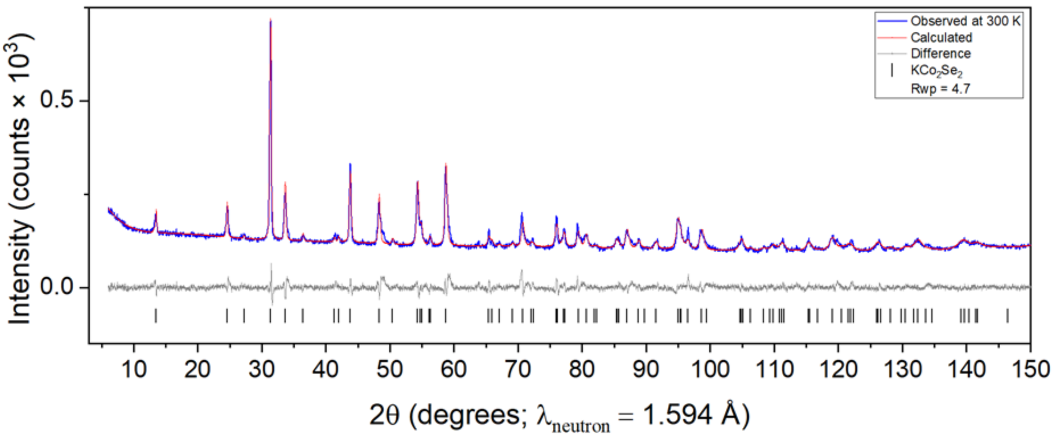

Figure S12. Rietveld refinement of KCo_2Se_2 against PND data measured at 300 K on D2B, R_{wp} = 4.7% and χ^2 = 1.67.

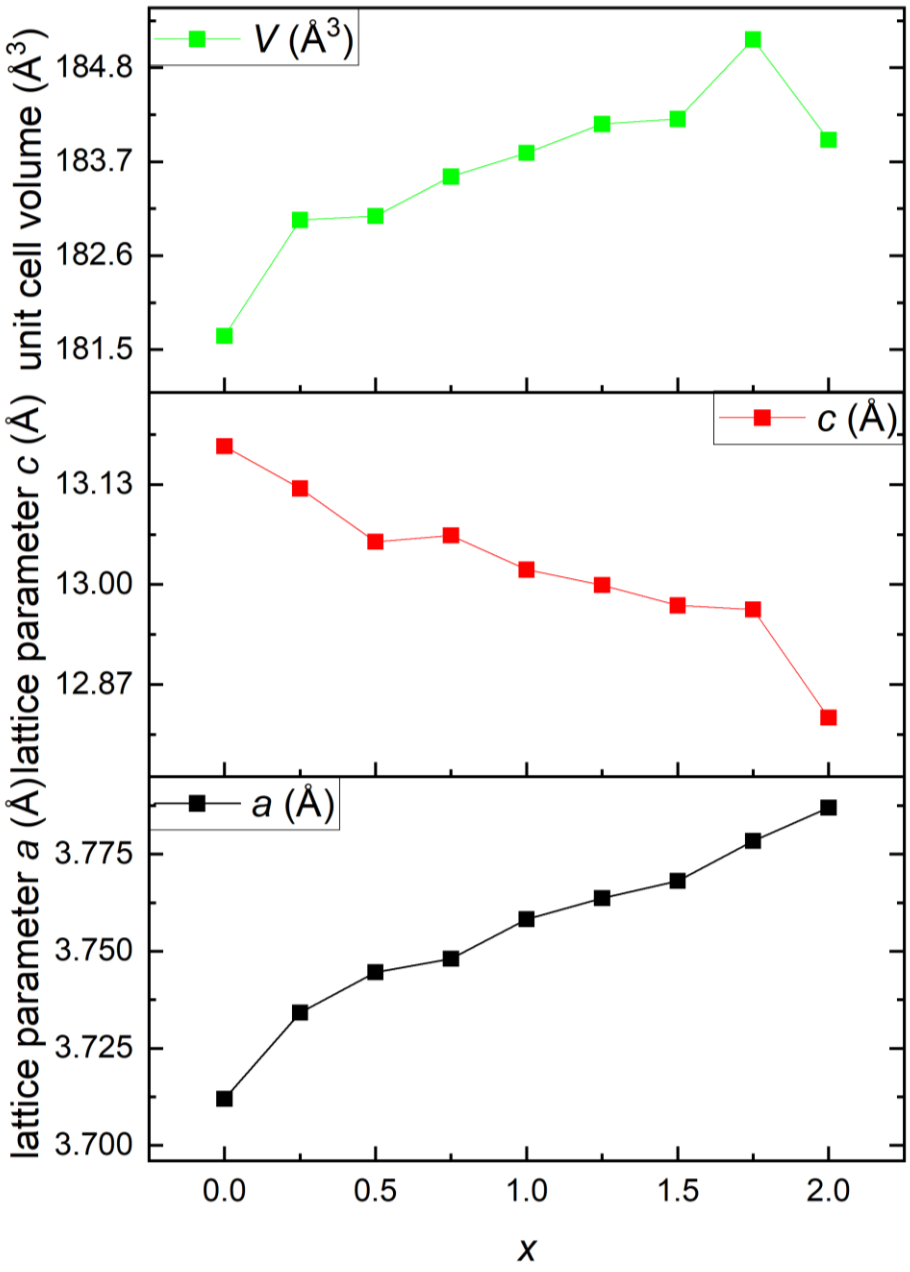

Figure S13. Changes in lattice parameters *a* and *c* and unit cell volume with *x* for $KCo_{2-x}Ni_xS_2$. The data was collected using the PSD detector at I11 (Diamond Light Source) and Rietveld refinements were performed on all members. The trend in lattice parameters with increasing nickel content (*x*) is not as smooth as for the selenide analogue. We attribute this to the greater sensitivity of the lattice parameters to slight sample displacement from the axis of the diffractometer when the PSD is used, compared with the case for the multi-analyser-crystal (MAC) detector used for the selenides where the use of analyser crystals reduces the sensitivity to the sample position.

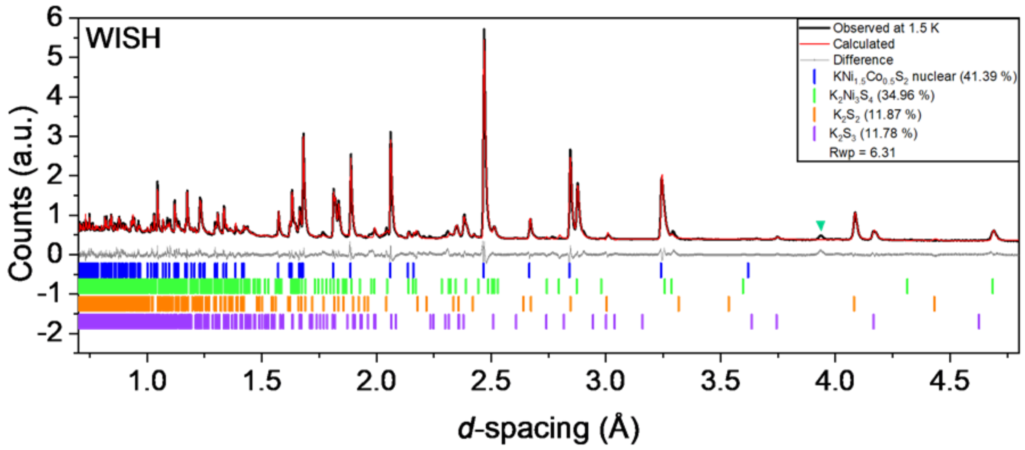

Figure S14. Rietveld refinement of $KCo_{0.5}Ni_{1.5}S_2$ against PND data measured at 1.5 K on WISH, R_{wp} = 6.31% and χ^2 = 1.04. Green triangle represents an unknown impurity.

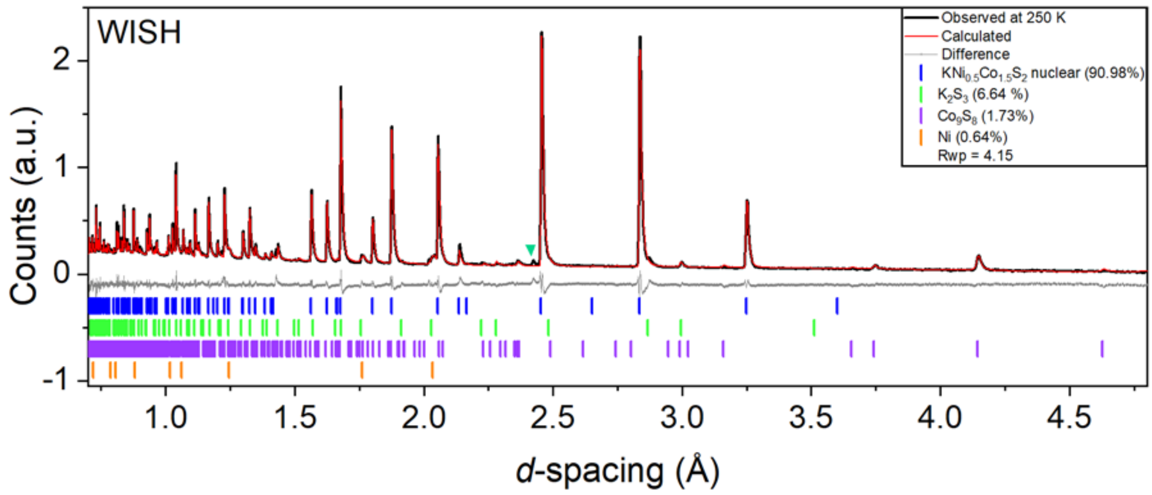

Figure S15. Rietveld refinement of $KCo_{1.5}Ni_{0.5}Se_2$ against PND data measured at 250 K on WISH, R_{wp} = 4.15% and χ^2 = 1.07. Green triangle represents an unknown impurity.

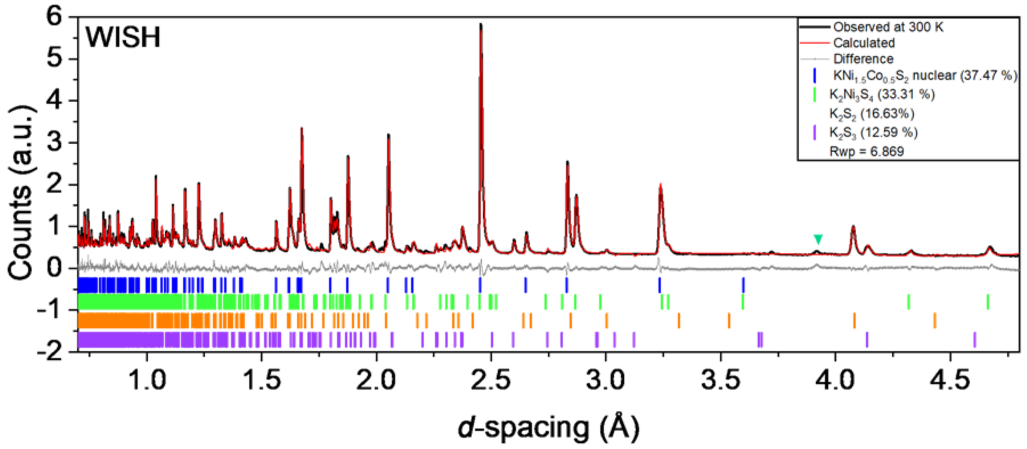

Figure S16. Rietveld refinement of $KCo_{0.5}Ni_{1.5}Se_2$ against PND data measured at 300 K on WISH, R_{wp} = 6.87% and χ^2 = 1.04. Green triangle represents an unknown impurity.

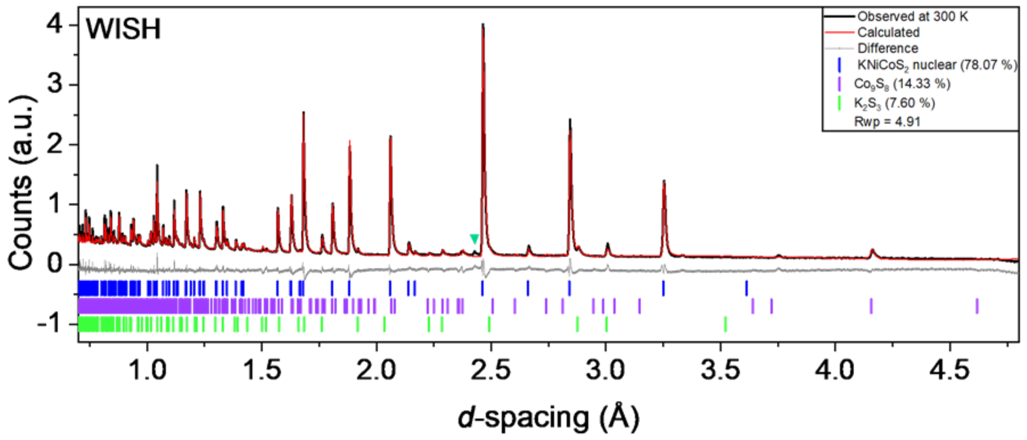

Figure S17. Rietveld refinement of $KCoNiS_2$ against PND data measured at 300 K on WISH, R_{wp} = 4.91% and χ^2 = 1.05. Green triangle represents an unknown impurity.

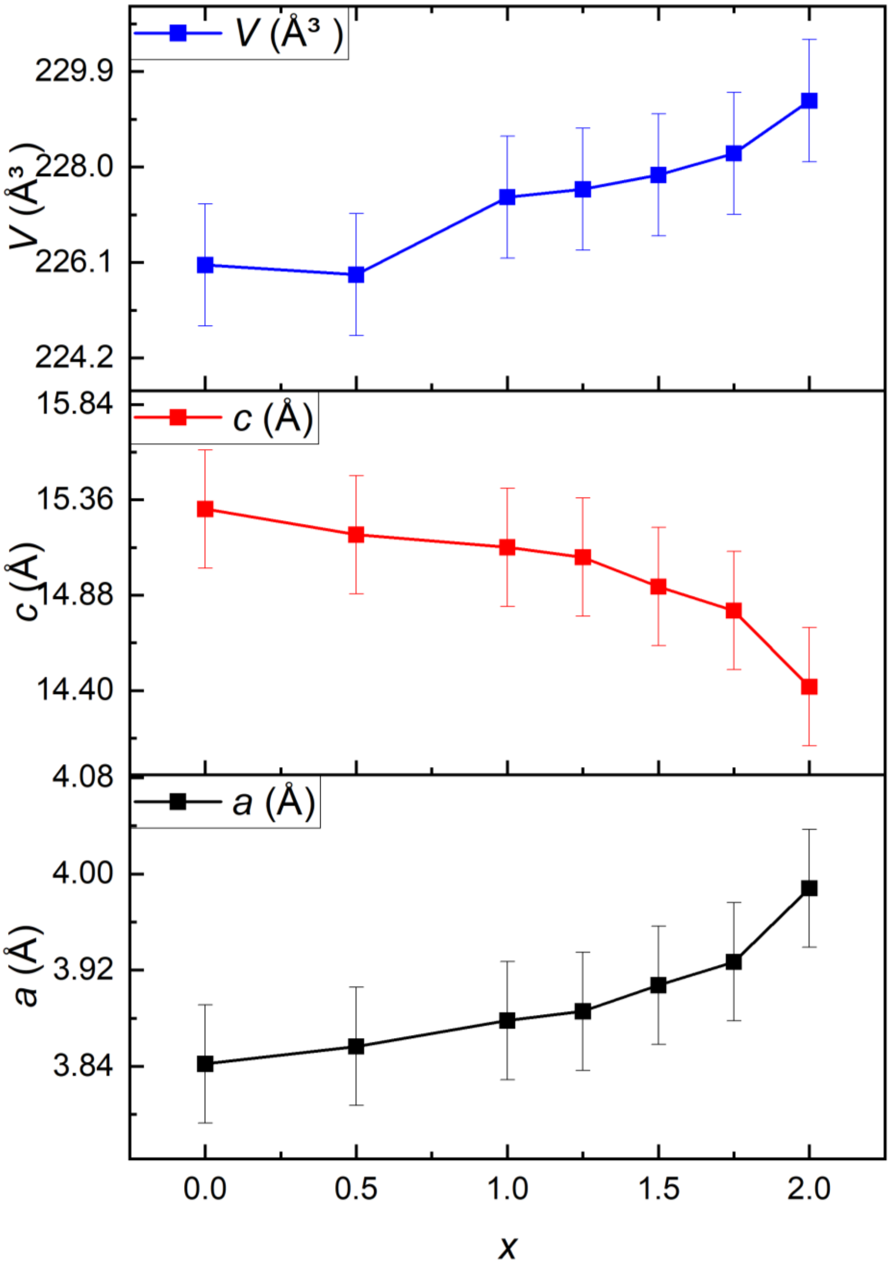

Figure S18. Changes in lattice parameters a and c and unit cell volume with x for $CsCo_{2-x}Ni_xSe_2$. The data was collected on the PSD detector at I11 (Diamond Light Source) and Rietveld refinements were performed on all members, except x = 0.25 and 0.75, as we have only collected the lab diffractometer data on the two members, rather than Synchrotron PXRD data (as for the rest). The data points corresponding to the series' end members – $CsCo_2Se_2$ (x = 0) and $CsNi_2Se_2$ (x = 2) are literature values.

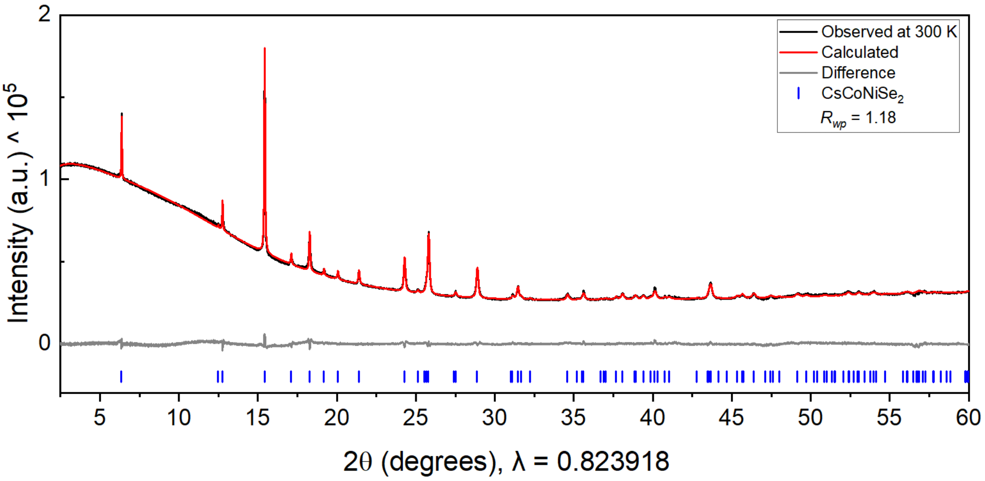

Figure S19. Rietveld refinement of $CsCoNiSe_2$ against Synchrotron PXRD data measured at 300 K on I11 at Diamond Light Source, R_{wp} = 1.18% and χ^2 = 2.43.

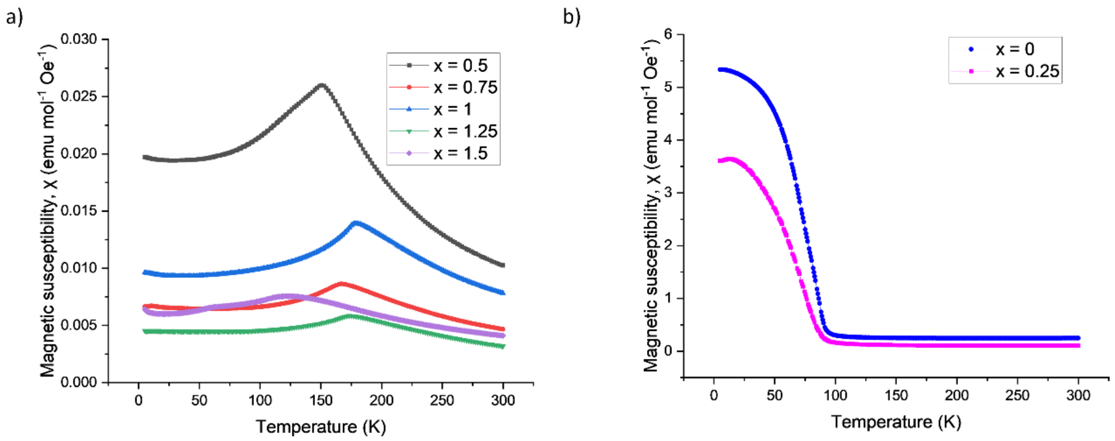

Figure S20: ZFC curves for a) antiferromagnetic members of $KCo_{2-x}Ni_xSe_2$ series (0.5 ≤ x ≤ 1.5), and b) ferromagnetic members of the series, i.e. KCo_2Se_2 (x = 0) and $KCo_{1.75}Ni_{0.25}Se_2$ (x = 0.25).
